# A novel serum spherical lectin from lamprey reveals a more efficient mechanism of immune initiation and regulation in jawless vertebrates

**DOI:** 10.1186/s11658-022-00401-0

**Published:** 2022-11-22

**Authors:** Jiali Lu, Jinsong Duan, Yinglun Han, Meng Gou, Jun Li, Qingwei Li, Yue Pang

**Affiliations:** 1 College of Life Sciences, Liaoning Normal University, Dalian, 116081 China; 2 Lamprey Research Center, Liaoning Normal University, Dalian, 116081 China; 3 State Key Laboratory of Membrane Biology, Beijing Advanced Innovation Center for Structural Biology, School of Life Sciences, Tsinghua University, Beijing, 100084 China

**Keywords:** Innate immunity, Lamprey, LSSL, Serum

## Abstract

The innate immune system is the body’s first line of defense against pathogens and involves antibody and complement system-mediated antigen removal. Immune-response-related complement molecules have been identified in lamprey, and the occurrence of innate immune response via the mannose-binding lectin-associated serine proteases of the lectin cascade has been reported. We have previously shown that lamprey (*Lampetra japonica*) serum can efficiently and specifically eliminate foreign pathogens. Therefore, we aimed to understand the immune mechanism of lamprey serum in this study. We identified and purified a novel spherical lectin (LSSL) from lamprey serum. LSSL had two structural calcium ions coordinated with conserved amino acids, as determined through cryogenic electron microscopy. LSSL showed high binding capacity with microbial and mammalian glycans and demonstrated agglutination activity against bacteria. Phylogenetic analysis revealed that LSSL was transferred from phage transposons to the lamprey genome via horizontal gene transfer. Furthermore, LSSL was associated with mannose-binding lectin-associated serine protease 1 and promoted the deposition of the C3 fragment on the surface of target cells upon binding. These results led us to conclude that LSSL initiates and regulates agglutination, resulting in exogenous pathogen and tumor cell eradication. Our observations will give a greater understanding of the origin and evolution of the complement system in higher vertebrates and lead to the identification of novel immune molecules and pathways for defense against pathogens and tumor cells.

**Supplementary Information:**

The online version contains supplementary material available at 10.1186/s11658-022-00401-0.

## Key points

- A novel serum spherical lectin from lamprey was identified which have agglutination activity against bacteria dependent on calcium.
- The crystal structure of LSSL was determined, and adhered to both microbial and mammalian glycans with high binding capacity.
- The role of LSSL was associated with MASP-1 and recruited C3 deposition for pathogen elimination.

**Supplementary Information:**

The online version contains supplementary material available at 10.1186/s11658-022-00401-0.

## Introduction

Bacteria produce many glycosylated structures and polysaccharides, including capsular polysaccharides, lipopolysaccharides (LPS), teichoic acid, and glycosylated proteins [1]. The glycan structure exposed on the surface of bacteria is the decisive factor in the interaction of bacteria with the external environment [2]. Exploring the interaction between pathogens and hosts has proven to be helpful for identifying pattern recognition receptors (PRRs), which can selectively interact with pathogen-associated molecular patterns (PAMPs) and contribute to the identification of bacteria [3]. As an important type of PRR, lectins play an important role in the host’s innate defense. Lectins can selectively target specific cells by recognizing glycan types and then mediate various biological processes from fertilization to pathogen clearance and immune response [4–7]. These lectins can be used for host defense, such as natural immune lectin and mannose-binding lectin (MBL) [8]. In the host serum, MBL is precomplexed with MBL-associated serine proteases (MASPs), and interaction of this complex with a cell surface results in activation of the lectin pathway of complements, ultimately leading to pathogen opsonization and clearance [9, 10]. Other humoral lectins implicated in immunity include ficolins, collectins, galectins, and HIP/PAP1 [11–13].

A group of lectins with unclear specificity is composed of intelectins (IntLs). The first IntL protein was reported in *Xenopus laevis* oocytes [14]. Homologs were subsequently found in other amphibians, fish, and many mammals. Following the detection of a closely related gene in Paneth cells in the small intestine of mice, this gene was named “intelectin” (intestinal lectin) [15]. Many vertebrate intelectin protein sequences are highly conserved, including eight cysteine residues with a fibrinogen-related domain (FReD) and an intelectin domain [16–18]. However, quaternary structure variations in various intelectins have been reported. For instance, mouse intelectin 1 is a monomer [19], while human intelectin-1 (hIntL-1) is a trimer [20]. Frog cortical granule lectin can form 9–12 multimers [21, 22], and ascidian intelectin exists in diverse multimers (including dimers, tetramers, hexamers, etc.) [23, 24].

IntL belongs to the lectin family called X-type lectin [16], which exists as a homopolymer consisting of 35 kDa monomers. They are reported to act as calcium-ion-dependent lectins. However, they do not contain the calcium-dependent C-type lectin sequence motif present in many human lectins [25]. In contrast, IntL contains a fibrinogen-like domain (FBD, residues 37–82 in hIntL-1), which is thought to be similar to fibrin, a type of innate immune lectin containing FBD [11, 26]. hIntL-1 does not bind to known human glycan epitopes, but interacts with terminal exocyclic 1,2-diols through calcium ions with multiple glycan epitopes found only in microorganisms. *N*-acetylneuraminic acid (Neu5Ac), a sialic acid that is abundant in human glycans, possesses an exocyclic 1,2-diol but does not bind hIntL-1, likely owing to unfavorable steric and electronic effects [27]. Intelectin plays a key role in maintaining the body’s own metabolism, immune regulation, and immune monitoring in jawed vertebrates. Our previous study demonstrated a homolog of the intelectin family protein in the lamprey serum [28]. Here, we demonstrate the structure of lamprey LSSL using cryogenic electron microscopy (cryo-EM) and how it mediates the immune mechanism of lamprey serum.

## Materials and methods

### Animals

Adult lampreys (*Lampetra japonica*) were captured from the Tong River of China and stored at 4 °C in a glass water tank. Adult lampreys (weight 200–220 g, length 49–52 cm) were anesthetized with 0.05% tricaine methanesulfonate (MS-222; 3-aminobenzoic acid ethyl ester, Sigma-Aldrich, St. Louis, MO, USA), and blood was collected from tail-severed lampreys and stored in a 2 mL centrifuge tube without coagulant at 4 °C.

### Negative staining of membrane and transmission electron microscopy

*Escherichia coli* (catalog number 43895, purchased from ATCC, Manassas, VI, USA), rabbit red blood cells (RRBCs from a rabbit purchased from the animal center at the Dalian Medical University), and HeLa cells (catalog number CCL-2, purchased from ATCC, Manassas, VI, USA) were pretreated for 1 h at 4 °C with 30% concentration of lamprey serum. Next, the sample was centrifuged at 2500*g* for 5 min and washed twice in 0.9% NaCl. The precipitate was resuspended in 1 mL PBS and 10% deoxycholate sodium (BBI Life Sciences, Montreal, Canada) and lysed via sonication with 30% power on ice by sonicator (SONICS-VCX150, Newtown, CT, USA). The total time for ultrasonication was 1 min, with a 5-s-on and 7-s-off cycle. The crude outer membranes were collected via centrifugation at 220,000*g* for 2 h at 4 °C (CP100NX ultracentrifuge; Hitachi, Tokyo, Japan). Next, the precipitate was resuspended in 150 μL 0.9% NaCl, added to carbon-coated grids (Zhongjingkeyi Technology, Beijing, China), negatively stained with 2% uranyl formate (AMEKO, Shanghai, China), and examined using transmission electron microscopy (TEM; JEM-2100Plus, Tokyo, Japan).

### Extraction of membrane constituents and analysis using mass spectroscopy

*Escherichia coli* (5 × 10^6^) were pretreated for 1 h at 4 °C with 30% concentration of lamprey serum. Membrane proteins were extracted as described above for TEM sample preparation and then analyzed via SDS–PAGE on a 12% gel. The protein strip was cut into gel and identified by mass spectrometry. The method is shown in the literature [29]. The obtained PMF-lift (Peptide Mass Fingerprinting) profiles were used to search for peptide matches using ProFound, which was run locally against our expression sequence tags (ESTs) in the lamprey cDNA library database.

### Purification of native LSSL using protein G affinity chromatography

First, 3 mL of the lamprey serum sample was diluted with 6 mL PBS. Next, the diluted lamprey serum was added to the Sepharose 4B affinity column (Sangon Biotech, Shanghai, China) at a flow rate of 0.5 mL/min in a nucleic acid/protein UV analyzer (HD-1, Huxi Analytical Instrument Factory, Shanghai, China) [28]. The column was washed with binding buffer (0.15 M NaCl, 20 mM Na_2_HPO_4_, pH 7.4), following which the elution buffer (0.1 M citric acid, pH 2.7) was added to the column at a rate of 0.5 mL/min until all the proteins were eluted. The pH of the eluted protein was adjusted using 1 M Tris–HCl (pH 8.0) until it stabilized at 7.0, following which the protein was dialyzed in 1× PBS. The eluted LSSL was detected using SDS–PAGE. The proteins were concentrated using a 10 kDa molecular weight cutoff (MWCO) ultrafiltration tube (Millipore Amicon Ultra, Boston, MA, USA), and the protein was concentrated to 2 μg/μL [28].

### Expression and purification of recombinant LSSL and preparation of antibody

The open reading frame of the *LSSL* gene was amplified and cloned into a pCold I vector with a histidine tag, and the signal peptide sequence was excised (MEASRLLLLLLLPLLLFCNSVAA). The purification of recombinant LSSL refers to the protein purification method in the article published previously [28]. The recombinant LSSL was expressed in Rosetta Blue competent cells induced by culturing in 1 mM isopropyl-1-thio-β-d-galactopyranoside (IPTG; Sangon Biotech) for 12 h at 16 °C. After 12 h of culture with shaking, the cells were harvested via centrifugation at 6000 rpm for 10 min at 4 °C. The induced cells were harvested and sonicated on ice until the sample was clear. The cell lysate was pelleted by centrifugation, and the soluble supernatant was collected for purifying the fusion protein, which was resuspended in binding buffer (20 mM Tris–HCl, pH 8.0), loaded onto a Ni–NTA His-Bind resin column (Novagen, USA) and washed with imidazole elution buffer (20 mM Tris–HCl, 150 mM NaCl, 20 mM imidazole, pH 8.0) at 1 mL/min. The purified LSSL was then examined using 12% SDS–PAGE and stained with Coomassie Brilliant Blue (Sangen Biotechnology). Balb/c mice were immunized with anti-LSSL polyclonal antibody prepared with recombinant LSSL as antigen. The antibody titer in the anti-LSSL serum was determined via ELISA at different dilutions, and the titer increased 640,000-fold from pre-immunization levels (pre-immunized mouse IgG was used as a negative control). The antibody was purified using a CNBr-activated Sepharose 4B column (GE Healthcare, Boston, MA, USA) according to the manufacturer’s instructions. The antibody specificity was confirmed using western blotting assays.

### Sequence, phylogeny, and synteny analyses

All amino acid sequences for the *Lethenteron reissneri* LSSL family members were obtained from a three-generation sequencing library, while the corresponding sequences for the other species were from the Ensemble (https://asia.ensembl.org/index.htmL) and NCBI (http://www.ncbi.nlm.nih.gov/) databases. The accession number is shown in Fig. 2A. SMART (http://smart.embl-heidelberg.de/) and PFAM (http://pfam.xfam.org/search) were used to predict the functional domains, and the online software SWISS-MODEL (https://swissmodel.expasy.org/interactive) was used to predict the three-dimensional structure of LSSL family genes. The MEME online software (http://meme-suite.org/tools/meme) was used to analyze the conserved amino acid sequences of the LSSL protein family, and 15 different motifs were identified. All collected amino acid sequences of the LSSL protein family from different species were input into Clustal X software, and then a phylogenetic tree was constructed using the MEGA 6.0 software by the neighbor-joining (NJ) method [30].

### Cryo-EM sample preparation and data acquisition

Four microliters (2 μg/μL) diluted protein samples were applied to the glow discharge ultra-flat porous carbon-coated TEM support grid (c-flat). After a 5-s waiting period, the grid was sucked dry for 3 s, and then quickly frozen in liquid nitrogen with Vitrobot Mark IV (Fei) at 100% humidity and 8 °C. The images were obtained on the Fei Titan Krios microscope, which operates at 300 kV with Fei Falcon II direct electron detector. AutoMATE II [31] was used at 75,000×. Automatic data acquisition was performed at nominal magnification, and a final pixel size of 1.30654 Å at the object scale was generated, with a defocus range of −1.5 to −2.5 μm. Each image was divided into 32 frames by dose, with a dose rate of about 1.5 e^–^s^−1^Å^−2^ and a total exposure time of 1 s. As mentioned above, spiral image analysis and three-dimensional image reconstruction were performed [32].

### Glycan chip assay

A 100-glycan array with eight-sample formats was used (Keruixin Biotechnology Co., Ltd, Nanjing, China). Subarrays were assayed with the LSSL protein, followed by incubation with mouse anti-LSSL primary antibody and goat anti-mouse IgG secondary antibody. Mouse IgG antibody purified from non-immunized mouse serum was used as the isotype control. After washing with washing buffer, the array slide was scanned with a LuxScan 10K microarray scanner (Bo’ao Instrument Co., Ltd., Suzhou, China) at 532 nm. There was no nonspecific binding in the print buffer. Mouse IgG was used as a positive control.

### Assaying glycan-binding of LSSL using ELISA

The glycan-binding assay was performed as described previously [33]. ELISA was performed to analyze the interaction between native LSSL proteins and microbial components. Briefly, 20 μg carbohydrates in 100 μL PBS (pH 9.6) per well was used to coat the ELISA plate for over 12 h at 4 °C. The plates were then blocked with 1% bovine serum albumin in PBS. LSSL (10–200 nM) was added to the plate and incubated at 37 °C for 1 h. The plate was then sequentially incubated with mouse-anti-LSSL (0.5 μg/μL, 1:100) primary antibody and horseradish peroxidase (HRP)-conjugated goat anti-mouse (1 μg/μL, 1:1000) secondary antibody at 37 °C for 1 h. After each step, the plate was washed with 0.5% Tween-20 in PBS four times. Finally, each well was incubated with 100 μL tetramethylbenzidine (TMB) for 15 min in the dark, and the reaction was stopped immediately using 100 μL of 2 M H_2_SO_4_. The absorbance was read at 450 nm. One representative experiment of three is shown. The background absorbance in samples without the protein was subtracted from the absorbance of the experimental samples.

### Determination of antibacterial activity

All supplies used in the antibacterial experiment were disinfected at 120 °C for 20 min using a high-pressure steam sterilization autoclave. The antibacterial experiments were conducted in a clean environment. Bacteria (*E. coli* and *S. aureus*) were first cultured overnight in a liquid medium containing 2.5 g peptone, 1.25 g yeast, 2.5 g NaCl, and 250 mL deionized water at pH 7.4–7.6 at 37 °C using an incubator shaker. After overnight shaking, the bacteria suspension was diluted 10^4^-fold with PBS. Then, 30% lamprey serum, LSSL-depleted serum (the native LSSL in lamprey serum was purified by Sepharose 4B and then concentrated by flow-through, which was the serum that depleted LSSL), and 100 μg/mL LSSL were mixed for 24 h, and PBS treatment group was used as blank control. Afterwards, 30 μL bacterial suspension was diluted to different concentrations (10^0^–10^6^) using PBS. Subsequently, 100 μL diluted bacterial suspension was carefully smeared on the agar plate, and the plates were incubated in an incubator at 37 °C for 24 h. Finally, the colonies were counted on the agar plate.

### Preparation of samples for scanning electron microscopy

*E. coli* and *S. aureus* (5 × 10^6^), grown to mid-log phase (OD_600_ 0.4–0.6), were incubated for 12 h with PBS, 30% concentration of lamprey serum, or LSSL (50 μg) at 4 °C. The bacteria were washed twice with PBS to remove debris and fixed overnight in 2.5% glutaraldehyde (Sigma-Aldrich) in 1× PBS at pH 7.4. The samples were washed with PBS and then dehydrated using an ascending ethanol gradient. The samples were mounted onto scanning electron microscopy (SEM) stubs, sputtered and plated with gold or chromium, and observed via DS130 SEM (ISI-TOPCON, Tokyo, Japan) using in-lens imaging.

### Bacterial agglutination assays using high-content screening

Bacterial agglutination due to LSSL binding was quantified. Fixed GFP-*E. coli* (Thermo Fisher Scientific, Waltham, MA, USA) suspension (OD_600_ 0.4–0.6) was treated with equal volumes of PBS, 30% concentration of lamprey serum, or LSSL (50 μg) for 12 h at 4 °C [33]. Next, the bacteria were washed twice with PBS to remove the debris. After incubation, the GFP-*E. coli* were plated in 96-well plates at a density of 10^5^ cells per well. The samples were analyzed using a high-content screening system per the manufacturer’s instructions (PerkinElmer, Waltham, MA, USA).

### Depletion of MASP-1 and C3 from serum

Cyanogen-bromide-activated Sepharose 4B (GE Healthcare, Boston, MA, USA) powder with cold 1 mM HCl and dissolved. The gel was filtered and then washed with distilled water. The filtrate was transferred to a solution of 0.1 M NaHCO_3_, pH 8.3, containing 0.5 M NaCl and the anti-MASP-1 monoclonal antibody (Sigma-Aldrich, St. Louis, MO, USA) or rabbit anti-lamprey-C3 polyclonal antibody [34] and placed under mild stirring for 3 h. The resulting suspension was washed thrice with 0.1 M sodium acetate, pH 4.0, plus 0.5 M NaCl, followed by 0.1 M Tris–HCl, pH 8.0, plus 0.5 M NaCl. At this time, the MASP-1 or C3 antibody was loaded onto a Sepharose 4B-Bind resin column (GE Healthcare, Boston, MA, USA). Three milliliters of the lamprey serum sample was diluted with 0.1 M Tris–HCl, pH 8.0, containing 0.5 M NaCl. Next, the diluted lamprey serum was added to the Sepharose 4B affinity column (Sangon Biotech, Shanghai, China) at a flow rate of 0.5 mL/min in a nucleic acid/protein UV analyzer (HD-1, Huxi Analytical Instrument Factory, Shanghai, China) [28]. The flow-through, i.e., the serum depleted in C3 or MASP-1, was collected immediately. The column was washed with binding buffer (0.15 M NaCl, 20 mM Na_2_HPO_4_, pH 7.4), following which the elution buffer (0.1 M citric acid, pH 2.7) was added to the column at a rate of 0.5 mL/min until all the proteins were eluted. The eluted proteins were detected using SDS–PAGE, and the depletion efficiency was verified by western blotting.

### Detection of molecular deposition on the cell surface

To detect the deposition of important immune molecules in the serum on the surface of the target cells, HeLa cells (5 × 10^6^) were treated with 30% concentration of serum, 30% concentration of depleted serum, or LSSL (50 μg) for 50 min at 4 °C, and then washed thrice with PBS. The cells were fixed with 4% paraformaldehyde (Sigma-Aldrich) in PBS for 15 min at room temperature, washed thrice with PBS, and then blocked with 5% donkey serum (Solarbio) in PBS for 1 h at room temperature [35]. Subsequently, the cells were incubated with mouse anti-polyclonal antibody (1 μg/μL, 1:1000) at 37 °C for 3 h and stained with FITC-labeled donkey anti-rabbit or mouse IgG (Thermo Fisher Scientific). The cells were stained with Hoechst (1:4000) (Thermo Fisher Scientific) for 5 min and then washed with PBS. Immunofluorescence was imaged with a Zeiss LSM 780 inverted microscope (Carl Zeiss, Jena, Germany Inc.) and analyzed using the Zeiss ZENLE software.

The same experimental samples were analyzed using flow cytometry. The flow cytometer was set at 488 nm (excitation wavelengths) to detect green fluorescence. Using PBS-treated cells as the negative control for flow cytometry correction, analyses were performed using the Modfit software. Three independent experiments were performed. Similarly, HeLa cells were treated under the above conditions, and the samples were prepared for western blotting analysis.

### Statistical analysis

All statistical analyses were performed using GraphPad Prism 8.0 software. Differences between treatment groups were determined using two-way analysis of variance (ANOVA). *P* < 0.05 was set as the threshold for significance (*P < 0.05, **P < 0.01, ***P < 0.001, ****P < 0.0001). Bar charts show the mean ± standard deviation (SD) of three independent experiments.

## Results

### Identification and purification of LSSL with spherical structures from lamprey serum

**Fig. 1:**
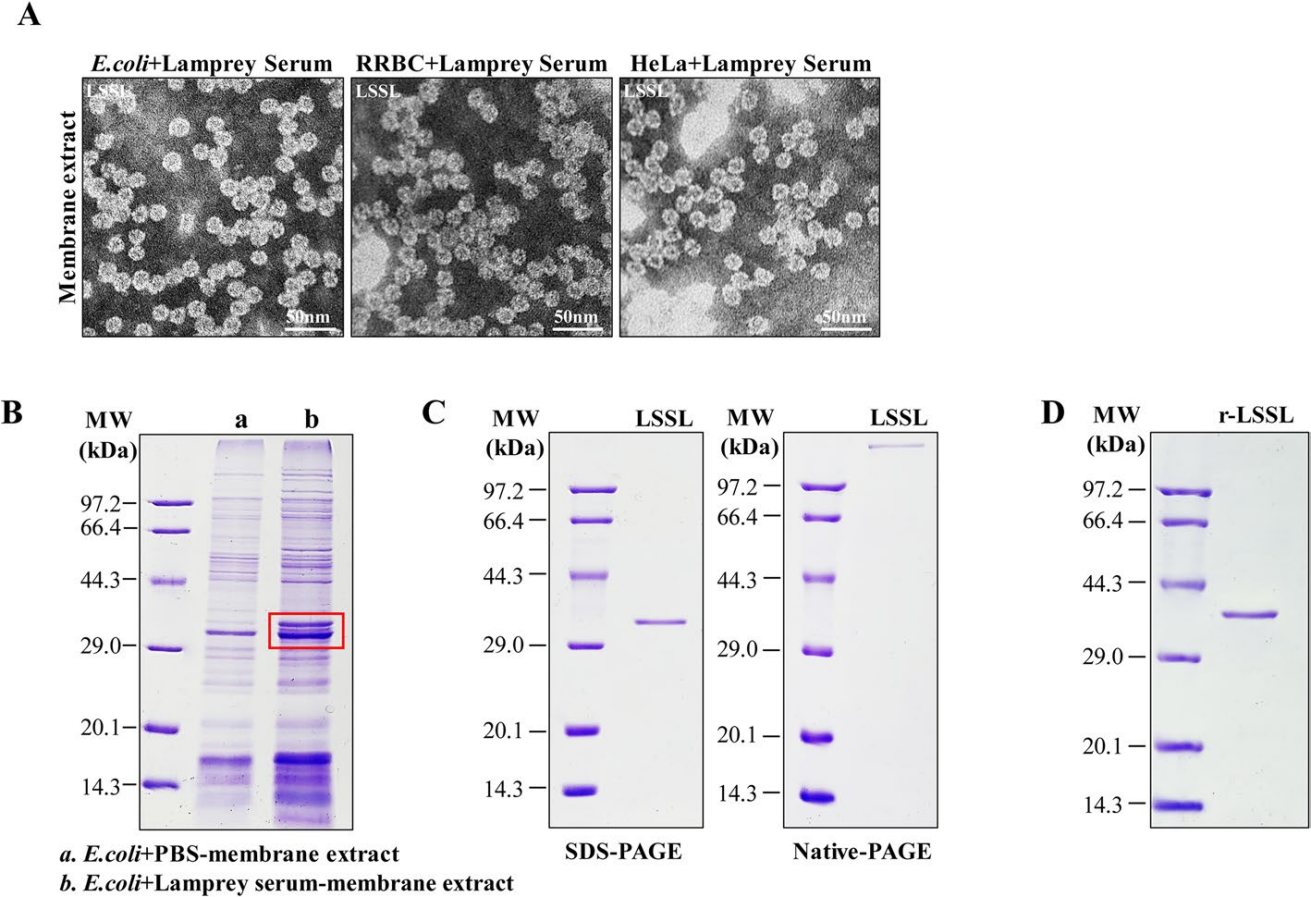
Identification of LSSL in lamprey serum. **A** Transmission electron microscopy (TEM) of membrane proteins from *Escherichia coli*, rabbit red blood cells (RRBCs), and HeLa cells treated with lamprey serum. Scale bar, 50 nm. **B** SDS–PAGE of membrane proteins extracted from *E. coli* treated with lamprey serum. The membrane proteins extracted from PBS-treated *E. coli* were used as the control. Protein bands (red box) were identified using mass spectrometry. **C** Detection of native LSSL protein purified from lamprey serum using SDS–PAGE and Native–PAGE. The molecular weight of the LSSL monomer is about 35 kDa. **D** SDS–PAGE of recombinant LSSL protein, the molecular weight of which is approximately 38 kDa

The experimental results revealed that membrane proteins were successfully extracted using ultracentrifugation from *E. coli*, RRBCs, and HeLa cells after treatment with the lamprey serum, following the traditional method of extracting human C9 [36]. Next, the extracted proteins were analyzed using TEM after negative staining with uranyl formate. A ring-shaped structure with an outer diameter of approximately 20 nm was observed in membrane extracts derived from all three cell types (Fig. 1A). To further identify the components of this structure, the proteins extracted from *E. coli* membrane treated with lamprey serum were subjected to SDS–PAGE. Compared with the normal *E. coli* membrane proteins without lamprey serum treatment, unique bands were observed in the serum-treated samples (Fig. 1B). Liquid chromatography–mass spectrometry (LC–MS) analysis of the target protein tryptic peptides revealed 13 unique peptides with > 95% probability (Additional file 1: Table S1). When these de novo peptide sequences were subjected to a BlastP search using the NCBI protein database, the MS sequence exhibited significant similarity with the known lamprey serum lectin (GenBank: BAB32787.1). However, we renamed the protein to lamprey serum spherical lectin (LSSL) on the basis of its structural characteristics (Additional file 2: Fig. S1A). Sepharose 4B affinity column was performed to purify native LSSL and produced a single band at approximately 35 kDa. As expected, LSSL migrated as a multimer on a non-reducing PAGE gel (Native–PAGE) (Fig. 1C). LC–MS analysis of the tryptic peptides of LSSL was performed to further confirm the identity of LSSL, which revealed 11 unique peptides with > 95% probability.

In addition, the full-length cDNA of LSSL was cloned into the pCold I vector, and recombinant LSSL was expressed as a histidine-tagged fusion protein in Rosetta Blue competent cells. The purified LSSL migrated as a single band with a molecular mass of approximately 38 kDa on a 12% SDS–PAGE gel (Fig. 1D). The purified flow-through was used as serum depleted of LSSL through Sepharose 4B affinity column for subsequent experiments. The 12% SDS–PAGE gel showed that there was almost no LSSL protein in the depleted fraction of LSSL compared with the serum group (Additional file 2: Fig. S1B). The 20-nm protein complexes were observed in the extracted cell membranes purified from LSSL-treated *E. coli*, RRBCs, and HeLa cells, similar to those observed in the serum-treated membrane preparations. Furthermore, this structure was not observed in the three cell types treated with LSSL-depleted lamprey serum (Additional file 2: Fig. S1C). We also found that both native and recombinant LSSL formed uniform spherical structures (Additional file 2: Fig. S1D). Atomic force microscopy further revealed that these dense spherical structure proteins were approximately 20 nm in diameter and 1.5 nm in thickness (Additional file 2: Fig. S1E). Finally, a mouse anti-LSSL polyclonal antibody was prepared to study the function of this proteins. The antibody titer reached 1:640,000 (Additional file 2: Fig. S1F), and western blotting further confirmed that the anti-LSSL antibody could specifically recognize and bind to LSSL in lamprey serum (Additional file 2: Fig. S1G).

*LSSL* contains FReDs consisting of 40–50 amino acids. To elucidate the origin and evolution of *LSSL*, a genetic phylogenetic tree was constructed on the basis of the NJ method (Additional file 3: Fig. S2A and Additional file 1: Table S2). The phylogenetic analysis shows that the lamprey *LSSL* gene is located outside the vertebrate *ITLN* (intelectin) family. The *LSSL* sequence was highly homologous to those of *ITLN1* and *ITLN2*. To analyze the conservation of *LSSL* genes, we identified 15 different motifs using the MEME motif analytical tool. Our results showed that the secondary structure of the LSSL molecule is relatively conserved. In addition to motifs 1 and 2 constituting FReD and motifs 7, 8, 9, and 10 constituting the intelectin domain, motifs 3, 4, 5, and 6 were present in almost all vertebrates and amphioxi, whereas motifs 11, 12, 13, 14, and 15 are present only in invertebrates. The intelectin domain does not exist in bacteriophages or invertebrates, while bacteria have motifs 8 and 9, which may represent the original prototype of the intelectin domain (Additional file 3: Fig. S2B). By combining the results of phylogenetic, domain, and motif analyses, we proposed a simple model for describing the evolution of *LSSL* genes. Specifically, the LSSL molecule may have originated from a phage with a unique FReD. Genes encoding phage proteins containing FReD may have then been incorporated into their host bacteria via lysogenic transformation. The bacterial gene would have subsequently integrated into the lamprey genome via transposition (Additional file 3: Fig. S2C).

### Cryo-EM structure of lamprey LSSL

**Fig. 2:**
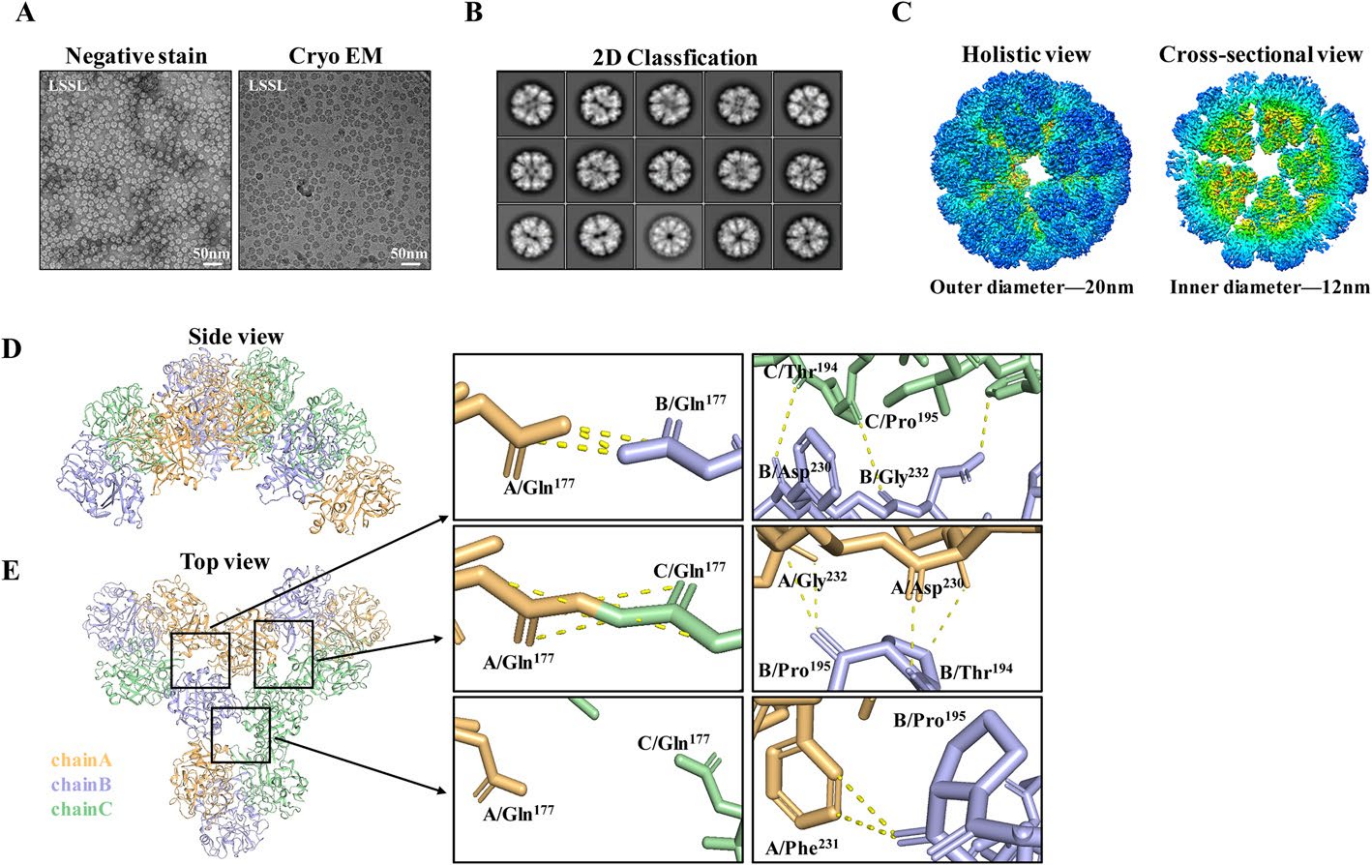
Structure and interactions of LSSL. **A** A representative negative stain (left) and cryo-electron microscopy (EM) (right) raw micrograph of LSSL. **B** Two-dimensional class averages of LSSL. **C** Surface representation of LSSL, viewed down the five-fold axis and colored by the radius (left). Cross-sectional view showing the internal features. LSSL assembled as icosahedral capsids, composed of incomplete symmetrical structure of five tetramers. Side view (**D**) and top view (**E**) of a tetramer structure. The interactions between tetramers in three orientations are shown in **E**. Orange, violet, and green indicate chain A, chain B, and chain C, respectively. The boxes indicate the magnified views of the interactions, and the representative residues are shown

The structural integrity of the native LSSL was examined using negative staining EM of the assembled homogeneous protein. The cryo-EM samples were imaged on a Titan Krios electron microscope using a Gatan K2 Summit detector (Fig. 2A). Approximately 500 single proteins from 1328 micrographs were manually picked, and two-dimensional (2D) classification was performed (Fig. 2B). Following five rounds of multi-reference 2D classification, 314,759 single proteins were selected for 3D classification. In total, 225,296 individual proteins were divided into four categories, and 37,328 proteins were used for the reconstruction of LSSL at an average resolution of 3.48 Å on the basis of the Fourier shell correlation (FSC) value of 0.143. A similar procedure on LSSL allowed the selection of 86,766 proteins, which yielded a structure exhibiting an average resolution of 3.34 Å (Additional file 4: Fig. S3A). LSSL possesses an oblong globular structure containing two highly twisted β sheet-containing structures surrounded by seven short α-helixes and extensive random coil regions (Additional file 4: Fig. S3B, C). The model exhibited good stereochemistry, and the quality of the final model was assessed using the statistics presented in Table 1. LSSL assembled as an icosahedral spherical structure with triangulation *T* = 4 that was composed of five incompletely symmetrical pentameric bodies. LSSL assembled homogeneously as approximately spherical hollow proteins of 20 nm diameter with a dense 12-nm-thick capsid layer (Fig. 2C). The structure of LSSL was determined at 3.3 Å resolution using the gold standard FSC criteria (Additional file 4: Fig. S3D).

**Table 1:** Data collection and refinement statistics for LSSL

Data collection	LSSL
EM equipment	Titan Krios (Thermo Fisher)
Voltage (kV)	300
Detector	Gatan K2 Summit
Energy filter	Gatan GIF Quantum, 20 eV
Pixel size (Å)	1.30654
Electron dose (e^−^Å^−2^)	50
Defocus range (µm)	~−1.5 to −2.5
Reconstruction software	RELION 3.1
Number of used particles	225,296
Symmetry	C1
Final masked resolution (Å)	3.344742
Map sharpening B-factor (Å2)	−137.37059
Model-building software PDB code	coot ID 7E1E

Additionally, the side view demonstrated that each trimer formed a unit, while three trimers were connected via face-sharing (Fig. 2D). On the basis of the top view of the structure, the homotrimer was divided into three single chains by color. The diagram on the right shows the interactions between chains within a distance of 3.5 Å. We observed that the contact between trimers was not completely symmetrical with three contact modes at Gln^177^ between the two adjacent chains. When two adjacent Gln^177^ were far apart (7.1 Å), the acyl group of the B chain Pro^195^ on the same side interacted with the benzene ring of the adjacent A chain Phe^231^. Meanwhile, two adjacent Gln^177^ from the A and C chains interacted with each other to form one side of a covalent bond. Furthermore, B chain Pro^195^ was found to be closer to A chain Gly^232^, with which it interacts. B chain Thr^194^ and A chain Asp^230^ were also detected on the adjacent chain. At the same time, an intermolecular interaction was observed between Tyr^229^ and Gly^232^ on the adjacent chain. Although an intermolecular interaction was observed between A chain Gln^177^ and B chain Gln^177^, a covalent bond was not formed. Similarly, we observed that C chain Thr^194^ and B chain Asp^230^ interacted with adjacent chains, while C chain Pro^195^ interacted with B chain Gly^232^ of the adjacent chain. These subtle differences in the interaction between the trimers may lead to nonsymmetrical icosahedral spherical structures (Fig. 2E).

### LSSL, hIntL-1, and *Xenopus laevis* embryonic epidermal lectin are structurally divergent

**Fig. 3:**
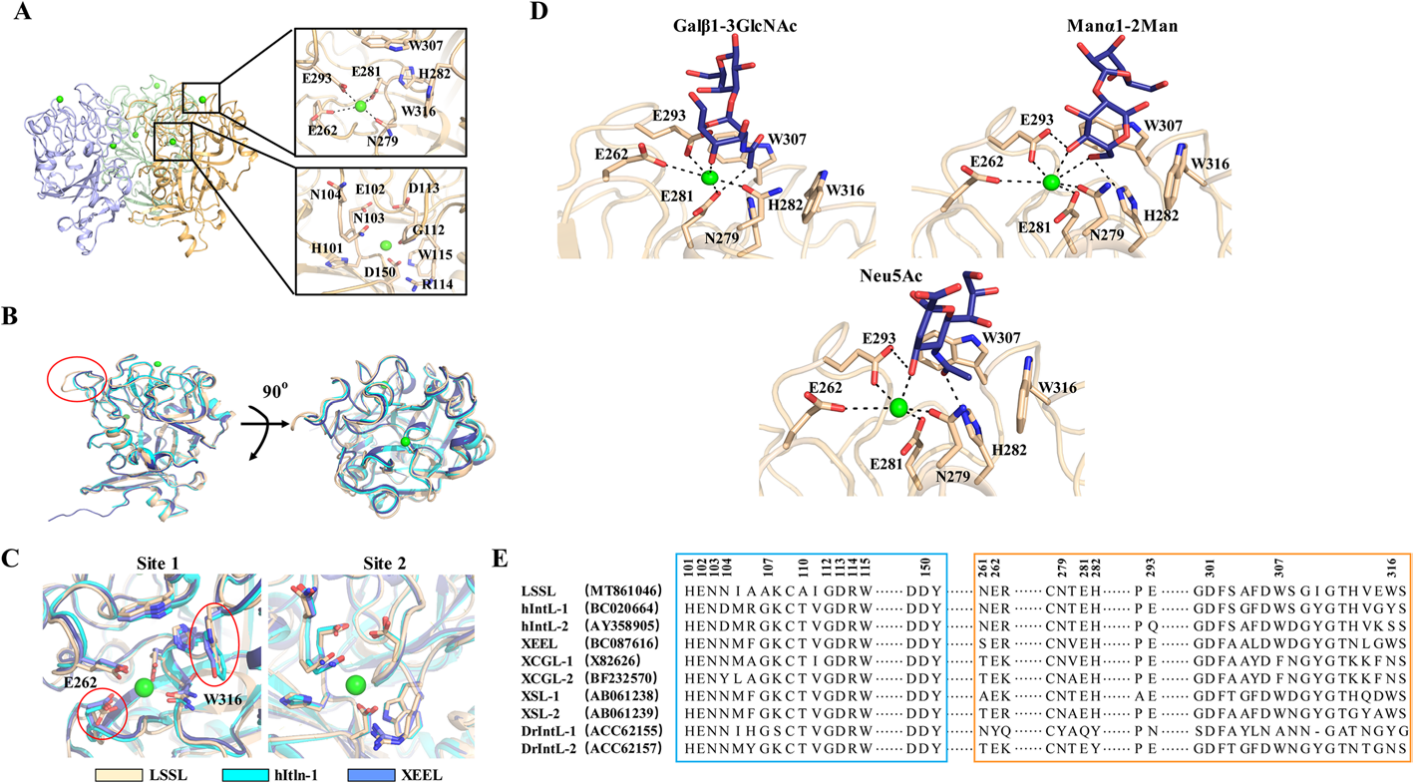
Comparative analysis of LSSL, hIntL-1, and XEEL. **A** Structure of the LSSL trimer with two calcium-ion-binding sites oriented with the intelectin-specific domains toward the top of and the fibrinogen-like lobes below the figure. Calcium site with two calcium ions (green) and no water molecules. The other calcium is buried deep in the protein core. **B**, **C** Comparison of the structure of lectins from lamprey (wheat), *Xenopus* (blue), and humans (cyan). The overall structure is shown in **B**. The Ca^2+^ binding site 1 and site 2 are shown in **C**. **D** Docking of Galβ1-3GlcNAc, Manα1-2Man, and Neu5Ac in the LSSL structure. The calcium ions are shown in green. **E** Sequence ratio pairs of XEEL and intelectins from humans [hIntL-1 (human intelectin-1) and hIntL-2 (human intelectin-2)] and common model organisms (XCGL-1, XCGL-2, XSL-1, and XSL-2 from *X. laevis*; mIntL-1 and mIntL-2 from mouse; DrIntL-1 and DrIntL-2 from zebrafish). The corresponding GenBank accession numbers are included in the second column. Residues in the structural calcium site and ligand-binding site are highlighted in blue (LSSL) and orange (hIntL-1/XEEL), respectively

LSSL is a disulfide-linked trimer in which each subunit was arranged around a crystallographic threefold axis, with two calcium-ion-binding sites (sites 1 and 2) in globular structures that do not belong to any well-known structural family. It has a fibrinogen-like lobe that contains a split and twisted seven-stranded β-sheet. The LSSL-specific region is characterized by a large fraction of random coil and a three-stranded curved β-sheet. The domains are encircled by 12 short solvent-exposed α-helical stretches (Fig. 3A). One calcium ion in site 2 is buried deep in the trimeric protein core where it is coordinated directly by the carboxylates of Asp^150^ (both oxygen atoms) and Asp^301^ and the backbone amide carbonyls of His^101^ and Gly^112^. It is presumed to play a structural role. Our results demonstrated that the amino acids Asn^261^ and Trp^316^ in site 1 are not conserved compared with the structures of hIntL-1 (PDB ID: 4WMY) [37] and *X. laevis* embryonic epidermal lectin (XEEL; PDB ID: 4WN0) [38]. The calcium ion in site 1 is directly coordinated by Glu^262^, Glu^281^, Glu^293^, and Asp^279^ located close to the protein surface, and the binding pocket geometry is determined by a network of 16 hydrogen bonds distributed among the four amino acids. However, LSSL does not contain any other calcium ion in the core and lacks water molecules in its structure, which distinguishes it from XEEL and hIntL-1. The comparative analysis of the position and size of the active pockets of the three proteins revealed that the carbohydrate recognition pocket was highly conserved. Furthermore, analogous to the carbohydrate binding site of hIntL-1 (PDB code: 4WMY), the hydroxyl groups of the disaccharides, namely, O(5) and O(6) in Manα1-2Man, O(5) in Galβ1-3GlcNAc and O(5) in Neu5Ac, serve as coordinating motifs for the surface accessible calcium ion. Additionally, the side chains in the carbohydrate-binding pocket of LSSL including Glu^262^, Glu^293^, and His^282^ are poised for hydrogen bonding, thereby enhancing calcium coordination. The carbohydrate vicinal exocyclic hydroxyl groups of the disaccharides adopt a gauche-like conformation as they chelate the calcium. Moreover, this portion of the disaccharides also fits well into a binding pocket formed by Trp^307^ and Trp^316^. The presence of these aromatic groups suggests that CH–π bonds may also contribute to the overall binding affinity. In addition, this pocket was located on site 1; hence, we predicted that site 1 might be critical for function. We also speculated that His^282^, Trp^307^, and Trp^316^ might participate in the interaction of the glycan but do not directly affect the calcium ions as they are far from the calcium ions (Fig. 3A).

In general, the overall structure of LSSL and the two calcium-binding sites are highly conserved compared with those of XEEL and hIntL-1. From a structural point of view, there are only three subtle differences. First, the loop of LSSL is longer than those of the others (Fig. 3B). According to its position in the polymer, this region may be involved in the interaction between protein molecules. Second, the side chain of Gln^262^ in the calcium-binding site is directed away from the center of the site. Such subtle differences may determine the binding with the substrate. Finally, LSSL and XEEL both contain Trp^316^, while hIntL-1 contains a tyrosine (Fig. 3C).

### Lamprey LSSL possesses high binding capacity for multiple ligands

To investigate the specificity of LSSL for saccharides, we applied LSSL to a glycan array containing 100 glycan structures on a glycan chip. A list of candidate structures with relative fluorescence units greater than 2000 obtained after screening against 8 μg/mL LSSL is presented in Additional file 5: Fig. S4A. Our results indicate that LSSL can bind to Galβ1-3GlcNAc, GlcNAcβ1-2Man, Manα1-2Man, and Neu5Ac. Furthermore, the preferred conformations of Galβ1-3GlcNAc, Manα1-2Man, and Neu5Ac that were compatible with LSSL binding were determined using modeling and computational methods (Fig. 3D). We aligned the sequences of intelectins from humans and several model organisms. As shown in Fig. 3E, the calcium-binding sites between hIntL-1 and XEEL were found to be highly conserved. The only difference was the sequence corresponding to Asn^118^ in XEEL. Most structural calcium residues were found to cluster between His^115^ and Asp^127^, with a HENXXXGXCTXGD consensus sequence. Although not present in the consensus sequence, Asp^162^ and Asp^311^ are also conserved residues of calcium-binding site. Meanwhile, the majority of the LSSL structural calcium site residue sequences are HENNXXXXXXXGDRW between 101 and 115 amino acids. In addition, these lectins comprise highly conserved amino acids in the ligand-binding region between Asn^261^ and Trp^316^, according to the crystal structure of XEEL_CRD_-GroP complex [38]. However, although highly homologous, a significant difference in glycan-binding abilities was observed between LSSL and other lectins. For instance, hIntL-1 cannot bind Neu5Ac via steric or electrostatic interactions [37].

### LSSL is involved in high-affinity molecular recognition and bacterial agglutination activity

**Fig. 4:**
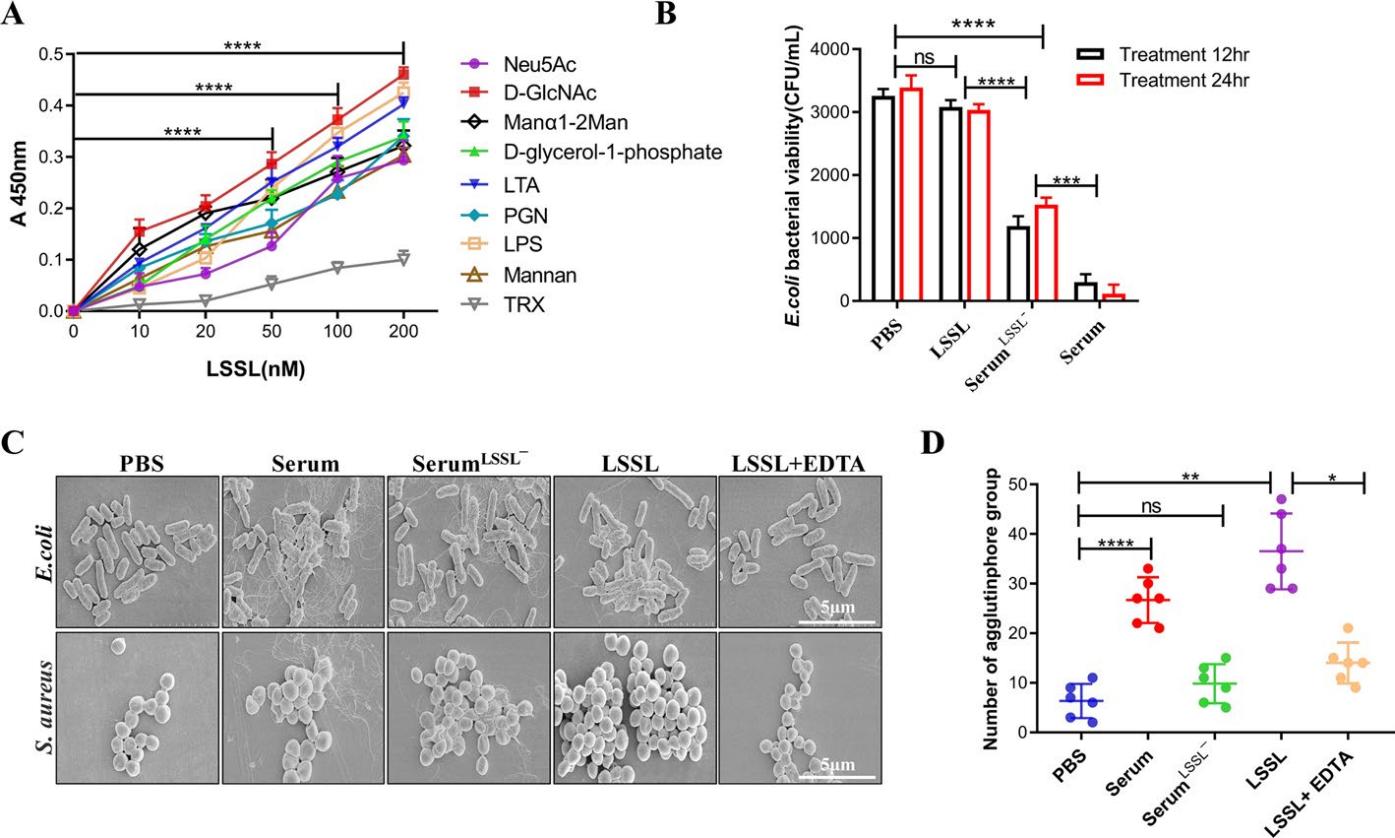
LSSL has strong bacterial agglutination effect. **A** ELISA showing the interaction between LSSL and microbial components and glycans. Plates were coated with 20 μg components, washed, and incubated overnight with LSSL at 4 °C, followed by detection using an anti-LSSL antibody. One representative experiment of three is shown (*n* = 3 technical replicates, data are representative of more than three independent experiments). Background absorbance without protein was subtracted. **B** The determination of bacterial activities of serum, LSSL, and LSSL-depleted serum on *E. coli* and *S. aureus* after 12 or 24 h. Data are presented as the mean percentage ± SD of three independent experiments. **C** Scanning electron microscopy (SEM) analysis of *E. coli* and *S. aureus* after treatment with LSSL. *E. coli* and *S. aureus* were incubated with PBS and used as controls. The concentration of EDTA used was 50 μg/mL. Scale bar, 5 µm. **D** Agglutination of GFP-*E. coli* by LSSL. Different components were incubated with FITC-labeled *E. coli* (10^5^ cells per well) in PBS for 1 h at room temperature and were examined using a high-content screening system (*n* = 3, ****P < 0.0001, ***P < 0.001, **P < 0.01, and *P < 0.05)

To further verify the binding of LSSL to glycans, the interaction between the LSSL and different glycan components was evaluated using ELISA. The results showed that LSSL could bind to Neu5Ac, D-GlcNAc, Manα1-2man, and d-glycerol-1-phosphate in a dose-dependent manner. This result is consistent with the results of the glycan chip assay. As expected, LSSL was also coordinated with terminal exocyclic 1,2-diols. In addition, LSSL bound to cell wall components of Gram-positive bacteria, such as lipoteichoic acid and peptidoglycan. It also bound strongly with LPS, an important component of the outer membrane of Gram-negative bacteria. LSSL also bound well with mannan, a polysaccharide derived from the yeast cell wall (Fig. 4A). As a negative control, thioredoxin did not bind significantly to LSSL. The deposition of Alexa 488-labeled LSSL on the surface of yeast cells corroborated the results of ELISA. Importantly, the deposition of LSSL on the cell membranes depended on calcium ions (Additional file 5: Fig. S4B).

To determine whether LSSL exerted a bactericidal effect, *E. coli* was treated with LSSL for 12 h and 24 h. As shown in Fig. 4B, lamprey serum has obvious bactericidal activity, while the bactericidal activity of LSSL did not vary significantly from that of the PBS-treated negative control group. When *E. coli* cells were treated with LSSL-depleted serum, the bactericidal activity improved significantly compared with that of the control serum group. Furthermore, *E. coli* or *S. aureus* were incubated with the recombinant LSSL proteins to determine the bacterial agglutination effect. The results of SEM showed that the recombinant LSSL caused significant agglutination of *E. coli* or *S. aureus* (Fig. 4C). Statistical analysis of the number of agglutinophore groups and the results of the fluorescence assay indicated that LSSL possesses stronger agglutination activity than serum (Fig. 4D and Additional file 5: Fig. S4C).

### Association of LSSL with MASP and C3 fragment deposition on the surface of target cells upon binding to pathogens

**Fig. 5:**
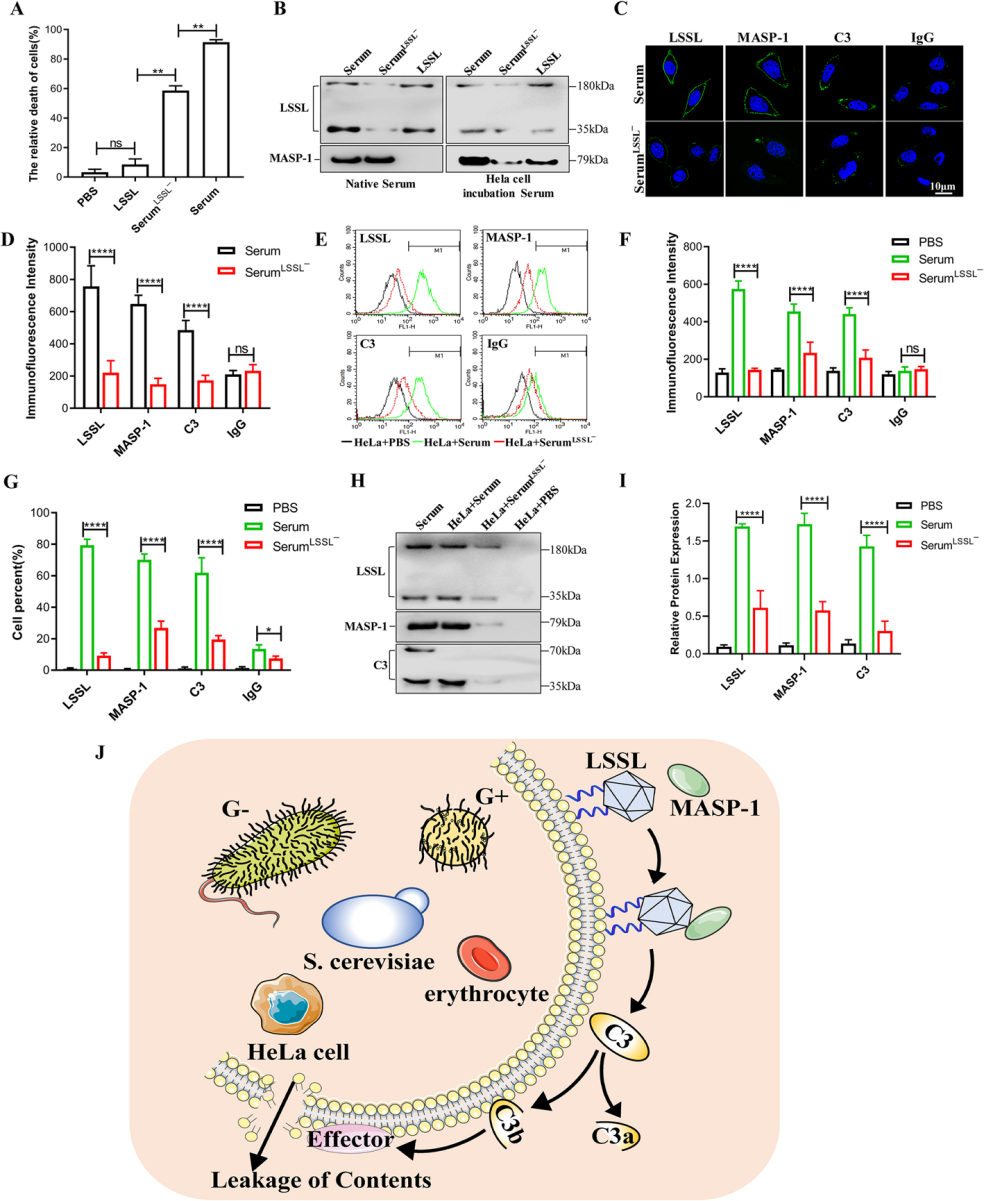
Analysis of LSSL-mediated innate immunity in lamprey serum. **A** Determination of cell activities elicited by 30% serum, 50 µg LSSL, and 30% LSSL-depleted serum on HeLa cells after 1 h. Data are presented as the mean percentage ± SD of three independent experiments. **B** Native LSSL was purified from serum when the HeLa cells were not pure, and the interaction between LSSL and MASP-1 was detected. **C** Immunofluorescence of LSSL, MASP-1, and C3 deposited on the surface of HeLa cells treated with lamprey serum and LSSL-depleted serum. Scale bars, 10 µm. IgG was used as the control. **D** Histogram showing the statistics of the above-mentioned results in terms of fluorescence intensity. The data are presented as the mean ± SD. **E** Quantitative analysis of the proteins on the cells using Alexa 488 staining, followed by flow cytometry. **F**, **G** Histogram showing the fluorescence intensity and cell percentage statistics of the above-mentioned flow cytometry results. The data are presented as the mean ± SD. **H** Western blotting analysis of the expression of LSSL, MASP-1, and C3 on HeLa cells using specific antibodies. Lamprey C3 is composed of three chains of α, β, and γ. We used β and γ chains to create rabbit polyclonal antibodies. The positions are shown in the figure. **I** Histogram showing the statistics of the western blotting results. All experiments were replicated at least three times, and similar results were obtained (*n* = 3, ****P < 0.0001, ***P < 0.001, **P < 0.01, and *P < 0.05). **J** In lampreys, LSSL recognizes glycans and activates MASP-1 to cleave C3. The active ingredient C3b recruits effector molecules to lyse pathogens

We next sought to determine whether LSSL exerts cytotoxic effects on eukaryotic cells. To this end, HeLa cells were treated with LSSL serum or LSSL-depleted serum for 12 h (Fig. 5A). LSSL had no cytotoxic effect on HeLa cells; however, the killing effect of LSSL-depleted serum was significantly decreased compared with normal serum, similar to the results previously observed in *E. coli*.

Furthermore, the interaction between LSSL and MASP in lamprey serum, which is homologous to mammalian MASPs, was investigated. To identify MASPs in the purified LSSL preparation, western blotting was performed using antibodies against MASP-1. However, no band was observed (Fig. 5B, left). Hence, we speculated that the association between LSSL and MASP-1 depends on their binding to pathogens or tumor cells. To verify this, lamprey serum was preincubated with HeLa cells, and LSSL was purified from serum using Sepharose 4B affinity column. The LSSL preparation was then subjected to SDS–PAGE and western blotting, and both LSSL and MASP-1 were detected with corresponding antibodies. This indicates that lamprey LSSL interacts with MASP-1 in the presence of pathogens or tumor cells (Fig. 5B, right).

Subsequently, we detected changes in MASP-1 upon depletion of LSSL from the serum. The results of immunofluorescence analysis clearly showed that the deposition of MASP-1 on the surface of HeLa cells was significantly reduced when LSSL was depleted from the serum using a specific antibody. The deposition of C3 on the surface of HeLa cells after the depletion of LSSL from the serum was significantly lower than when the cells were treated with native serum (Fig. 5C, D). Similar results were obtained using flow cytometry (Fig. 5E–G) and western blotting (Fig. 5H, I). To further confirm the relationship between LSSL, MASP-1, and C3, MASP-1 and C3 molecules from serum were predepleted using the corresponding specific antibodies. The results of flow cytometry and western blotting showed that MASP-1 depletion of serum did not significantly affect LSSL deposition on the surface of HeLa cells. However, the deposition of C3 molecules on the surface of HeLa cells treated with the MASP-1-depleted serum was significantly reduced according to the flow cytometry and western blotting results, indicating that MASP-1 directly affects the activation of C3 (Additional file 6: Fig. S5A–E). We also observed that the deposition of LSSL and MASP-1 on the surface of HeLa cells incubated with the C3-depleted serum did not change (Additional file 7: Fig. S6A–E). On the basis of the above results, we concluded that the novel lectin, LSSL, might play a pivotal role in the recognition of pathogens and subsequent activation of the complement system.

## Discussion

In the present study, the native LSSL from lamprey serum was first purified, and the structural characteristics were analyzed using cryo-EM and MS. We demonstrated that lamprey possessed a distinctive complement pathway activated by LSSL. Moreover, the LSSL sequence was found to be highly homologous with serum lectin and intelectin, which contain FReD and an intelectin domain.

The crystal structure of the LSSL monomer was compared with those of XEEL_CRD_ and hIntL-1; the results revealed that the LSSL monomer contains only two calcium ions, in contrast to hIntL-1 and XEEL_CRD_, which contain three [37, 38]. Analysis of the amino acids situated near the calcium ions revealed that Asn^261^ and Trp^316^ were not conserved in hIntL-1 and XEEL_CRD_. In addition, the position and size of the active pockets of the three proteins were identical and highly conserved. Considering that this pocket was located in site 1, we predicted that site 1 might be critical for LSSL function. Moreover, LSSL requires calcium ions to perform its biological function, which has been confirmed in this study, consistent with the recognition of pathogen-specific carbohydrates by intelectins in a Ca^2+^-dependent manner [15, 16, 26]. Although intelectins are not members of the C-type lectin family, they also use calcium ions to coordinate with their carbohydrate ligands [17, 20]. LSSL and other intelectins share > 80% sequence similarity, including a putative conserved FReD. FReD is not unique to LSSL, as it is also found in other proteins such as ficolin lectin, indicating that the same domain may play different functions. However, although ficolin lectin is involved in sugar recognition via FReD, the sequences of FReD of ficolin and LSSL may differ [39, 40]. LSSL contains not only FReD but also an intelectin domain of 100 amino acids at the C-terminus of LSSL.

Next, we investigated the origin and evolution of *LSSL*. On the basis of comparative genomics and phylogenetic analyses, we proposed a simple model describing the evolution of intelectin genes. Molecular evolution traced back to a gene encoding the *Pseudomonas* phage protein with a unique FReD in the phage genome, which was possibly integrated into the host *Pseudomonas* genome via gene fusion and subsequently transferred into the bacterial genome via horizontal gene transfer, resulting in an early intelectin domain. Subsequently, this gene was transferred from bacteria to the lamprey genome through a transposable locus to form the *LSSL* gene, which encodes the intelectin domain in lampreys. Moreover, LSSL comprises of a trimer as its subunit, which self-assembles into an icosahedral globular structure with a diameter of 20 nm, similar to the structure of a virus. Proteins with similar icosahedral structures, such as Arc proteins [32] and IMEF cargo proteins [41], have been reported previously. *Arc*, a neuronal gene, originated via the domestication of the retrotransposon *Gag* and transferred RNA in cells within virus-like capsids [32]. The IMEF encapsulin self-assembles into a 240-subunit icosahedral compartment with a large iron storage capacity [41]. Therefore, we speculated that LSSL with an icosahedral spherical structure might provide a larger region for binding to the sugar chain on the surface of the pathogen, increasing the extent of binding to the pathogen, thereby promoting cell or bacterial agglutination. As shown in Fig. 4, LSSL was strongly involved in extracellular bacterial recognition and agglutination.

It is well known that the complement system is activated via the classical pathway, alternative pathway, and lectin pathway, followed by the formation of the membrane attack complex, finally leading to the elimination of pathogens [42]. The lectin pathway involves carbohydrate recognition by MBL and the subsequent activation of MASPs and C3; similar complement molecules of the lectin-based complement pathway also exist in lampreys [43] and ascidians [44]. Recently, human ficolins in serum were found to associate with MASPs and activate the lectin pathway [45]. Here, we confirmed that LSSL and MASP interact to activate C3 molecules and exert immune functions in the presence of pathogens or tumor cells. In fact, several innate immune molecules from lamprey serum such as MBL [46], C1q [47], and LIP [48, 49] are involved in recognition and host defense against various pathogens. Among these proteins, the LSSL content in serum is relatively higher, with approximately 500 μg of LSSL separated and purified from 3 mL of serum in the present study, whereas the serum content of LIP was negligible (240 ng/mL serum) and difficult to detect using western blotting. Our results confirmed that the removal of LSSL protein from serum directly affected MASP recruitment and C3 deposition, and that the MBL- and C1q-mediated immune pathways were weaker than that mediated by LSSL. As demonstrated in Fig. 5, the deposition of MASP-1 and C3 on the target cell surface was significantly reduced, and the cytocidal effects on target cells were decreased after LSSL was depleted from serum. Therefore, the LSSL-mediated immune pathway might be involved in the initiation and regulation of immune responses in lampreys (Fig. 5J). In addition, we previously demonstrated that variable lymphocyte receptor B (VLRB), in collaboration with C1q, activates C3 and mediates complement-dependent cytotoxicity [34]. Surprisingly, the deposition of VLRB and C3 on the surface of target cells following depletion of LSSL from serum increased significantly, compared with that after treatment with normal serum (Additional file 8: Fig. S7A–G), indicating that LSSL first binds to the pathogen before performing an immune function in lamprey serum, while the VLRB-activated response is primarily associated with adaptive immune function. Our collective findings suggest that the LSSL-mediated immune pathway plays a vital role in pathogen recognition and initiation of immune responses. The immune pathway mediated by LSSL is different from the complement system in higher vertebrates and is a specialized form of the innate immune pathway in lamprey serum.

In conclusion, our results demonstrated that the spherical structural properties of LSSL are evolutionarily conserved and may provide a structural basis for understanding their function in the future. Moreover, LSSL showed broad-spectrum sugar-recognizing properties, recruited MASP, and activated C3, which are molecules involved in innate immune responses.

## Supplementary Information

**Additional file 1: Table S1.** LC–MS analysis of tryptic-digested peptides of LSSL. **Table S2.** The full names and abbreviation of species used in the analysis.

**Additional file 2: Fig S1.** Discovery of LSSL in lamprey serum. (A) Amino acid sequences of lamprey LSSL. The FReD domain is shown in red and the Intelectin domain is shown in blue. (B) Detection of depletion of LSSL protein in lamprey serum by SDS-PAGE. (C) TEM of membrane protein from *E. coli*, RRBCs, and HeLa cells treated with LSSL and LSSL-depleted serum. Scale bar, 50 nm. (D) Detection of native and recombinant LSSL protein by TEM. Scale bar, 50 nm. (E) The structure of LSSL as determined by atomic force microscopy. The measurement results show a diameter of 20 nm and a height of 1.5 nm. (F, G) Titer detection of LSSL polyclonal antibodies and detection of specificity by western bloting.

**Additional file 3: Fig S2.** Origin and evolution of *LSSL*. (A) The phylogenetic tree for the LSSL family based on the neighbor-joining (NJ) method. An NJ tree was constructed using the amino acid sequences of the LSSL proteins. All species represented by abbreviations are shown in Table S2. The bar (0.020) indicates the genetic distance. Different color lines correspond to different species groups. (B) The motif composition of the LSSL family proteins. (C) Model diagram showing the mechanism of LSSL evolution.

**Additional file 4: Fig S3.** Cryo-EM analysis. (A) A flow-chart of cryo-EM data processing. (B) Subunit structure in LSSL trimer. (C) Fold diagram for the structure of LSSL. (D) FSC curve (gold standard FSC) as a function of before and after mask application.

**Additional file 5: Fig S4.** Glycan selectivity of LSSL assessed by glycan microarrays. (A) LSSL (8 µg/mL) binding to 100 glycan microarray. The concentrations given for the glycan microarray represent those used in the carbohydrate immobilization reaction. Data are presented as the mean ± SDs; (*n* = 3 technical replicates). (B) Confocal microscopy detection of LSSL binding to yeast. LSSL were incubated with yeast cells (10^5^ cells/well) in PBS for 1 h at room temperature and incubated with FITC-conjugated anti-mouse IgG secondary antibodies followed by confocal microscopy analysis. (C) Quantitative statistical results of GFP-*E. coli* after high-content screening of bacterial agglutination after LSSL treatment. Bacteria were observed using high-content screening and photographed at the indicated time points (40× magnification).

**Additional file 6: Fig S5.** Elimination of MASP-1 from serum to detect the deposition of LSSL and C3 on the surface of Hela cells. (A) Quantitative analysis of proteins on HeLa cell membranes analyzed by Alexa-488 staining followed by flow cytometry. (B, C) The histogram shows the fluorescence intensity and cell proportions of the above flow cytometry results, respectively. The data are presented as the means ± SDs. (D) Western blotting analysis of depleted and undepleted MASP-1 serum treated HeLa cells, the LSSL, MASP-1, and C3 expression using specific antibodies. (E) Histogram presenting the statistics of the western blotting results. All experiments were repeated at least three times with similar results (*n* = 3, ****P < 0.0001 ***P < 0.001 **P < 0.01 and *P < 0.05).

**Additional file 7: Fig S6.** Elimination of C3 from serum to detect the deposition of LSSL and MASP-1 on the surface of HeLa cells. (A) Quantitative analysis of proteins on the HeLa cells analyzed by Alexa-488 staining followed by flow cytometry. (B, C) The histogram shows the fluorescence intensity and cell proportions of the above flow cytometry results, respectively. The data are presented as the means ± SDs. (D) Western blotting analysis of depleted and undepleted C3 serum treated HeLa cells, the LSSL, MASP-1, and C3 protein expression using specific antibodies. (E) A histogram showing the statistics of the western blotting results. All experiments were repeated at least three times with similar results (*n* = 3, ****P < 0.0001 ***P < 0.001 **P < 0.01 and *P < 0.05).

**Additional file 8: Fig S7.** Elimination of LSSL from serum to detect the deposition of VLRB and C1q on the surface of HeLa cells. (A) Immuno-fluorescence detection of VLRB and C1q deposited on the surface of HeLa cells treated with lamprey serum and LSSL-depleted serum. Scale bars, 10 µm. (B) A histogram shows the statistics of the above-mentioned results. The data are presented as the means ± SDs. (C) Western blotting analysis with mouse anti-VLRB monoclonal antibodies [34] and rabbit anti-C1q [34]. (D) A histogram showing the statistics of the western blotting results. All experiments were repeated at least three times with similar results. (E) Quantitative analysis of proteins on the cell analyzed by Alexa-488 staining followed by flow cytometry. (F, G) The histogram shows the fluorescence intensity and cell percentage statistics of the above flow cytometry results, respectively. The data are presented as the means ± SDs (*n* = 3, ****P < 0.0001 ***P < 0.001 **P < 0.01 and *P < 0.05).

## Data Availability

The raw data supporting the conclusions of this article will be made available by the authors, without undue reservation, to any qualified researcher.

## Abbreviations

VLR: Variable lymphocyte receptors
LSSL: Lamprey serum spherical lectin
LPS: Lipopolysaccharides
PRRs: Pattern recognition receptors
PAMPs: Pathogen-associated molecular patterns
MBL: Mannose-binding lectin
MASP: MBL-associated serine proteases
IntLs: Intelectins
FReD: Fibrinogen-related domain
hIntL-1: Human intelectin-1
β-Galf: β-Linked d-galactofuranose
FBD: Fibrinogen-like domain
KO: d-Glycero-d-talo-oct-2-ulosonic acid
KDO: 3-Deoxy-d-manno-oct-2-ulosonic acid
Neu5Ac: *N*-acetylneuraminic acid
cryo-EM: Cryogenic electron microscopy
ESTs: Expression sequence tags
IPTG: Isopropyl-1-thio-β-d-galactopyranoside
SEM: Scanning electron microscopy
XEEL: *X. laevis* embryonic epidermal lectin
VLRB: Variable lymphocyte receptor B
