# Supplementary material for: A novel serum spherical lectin from lamprey reveals a more efficient mechanism of immune initiation and regulation in jawless vertebrates
Source: Cell Mol Biol Lett. 2022 Nov 22;27:102. doi: 10.1186/s11658-022-00401-0 (PMC9682848; doi:10.1186/s11658-022-00401-0)
Supplement: Supplementary file 1 — Additional file 1: Table S1. LC–MS analysis of tryptic-digested peptides of LSSL. Table S2. The full names and abbreviation of species used in the analysis. [file 11658_2022_401_MOESM1_ESM.docx]

| **MH+[Da]** | **RT[min]** | **Sequence** | |
| --- | --- | --- | --- |
| 1142.4884 | 21.46 | | VESATDDDYK |
| 1670.8112 | 23.5 | | TKTKEDGVYcLQTK |
| 1212.5609 | 27.21 | | EDGVYcLQTK |
| 2750.2349 | 37.19 | | VLDNcNTEHYcIGGAGYVPEQTPR |
| 1091.5699 | 37.87 | | APFALcSGLR |
| 1165.5804 | 41.87 | | SWANLNTFGR |
| 3503.5936 | 53.31 | | SGQFYQAFcDmNTNGGGWTLVASVHENNIAAK |
| 2572.1317 | 57.01 | | QcGDFSAFDWSGIGTHVEWSASK |
| 3487.5926 | 57.27 | | SGQFYQAFcDMNTNGGGWTLVASVHENNIAAK |
| 2348.1739 | 61.24 | | YHTATEFLTPLGGNLYFLYK |
| 4178.9356 | 62.47 | | VESATDDDYKNPGYFDVDAEDISVWHVPNGTPLAQWK |
| 3055.4767 | 65.24 | | NPGYFDVDAEDISVWHVPNGTPLAQWK |
| 1428.7983 | 69.54 | | SLLEAAVFIFYR |

**Table S1. LC-MS analysis of tryptic-digested peptides of LSSL**

**Table S2. The full names and abbreviation of species used in the analysis**

| **Species** | **Abbreviation** |
| --- | --- |
| *Homo sapiens* | Hs |
| *Mus musculus* | Mm |
| *Xenopus tropicalis* | Xt |
| *Danio rerio* | Dr |
| *Poecilia formosa* | Pf |
| *Poecilia latipinna* | Pl |
| *Lampetra japonica* | Lj |
| *Lethenteron reissneri* | Lr |
| *Branchiostoma belcheri* | Bb |
| *Deltaproteobacteria bacterium* | Db |
| *Rickettsiales bacterium* | Rb |
| *Stylophora pistillata* | Sp |
| *Pocillopora damicornis* | Pd |
| *Exaiptasia pallida* | Ep |
| *Synechococcus phage* | Syn |
| Cyanophage | Cya |
| *Cotesia plutellae polydnavirus* | Cp |
