# Supplementary figures and images for: A novel serum spherical lectin from lamprey reveals a more efficient mechanism of immune initiation and regulation in jawless vertebrates
Source: Cell Mol Biol Lett. 2022 Nov 22;27:102. doi: 10.1186/s11658-022-00401-0 (PMC9682848; doi:10.1186/s11658-022-00401-0)

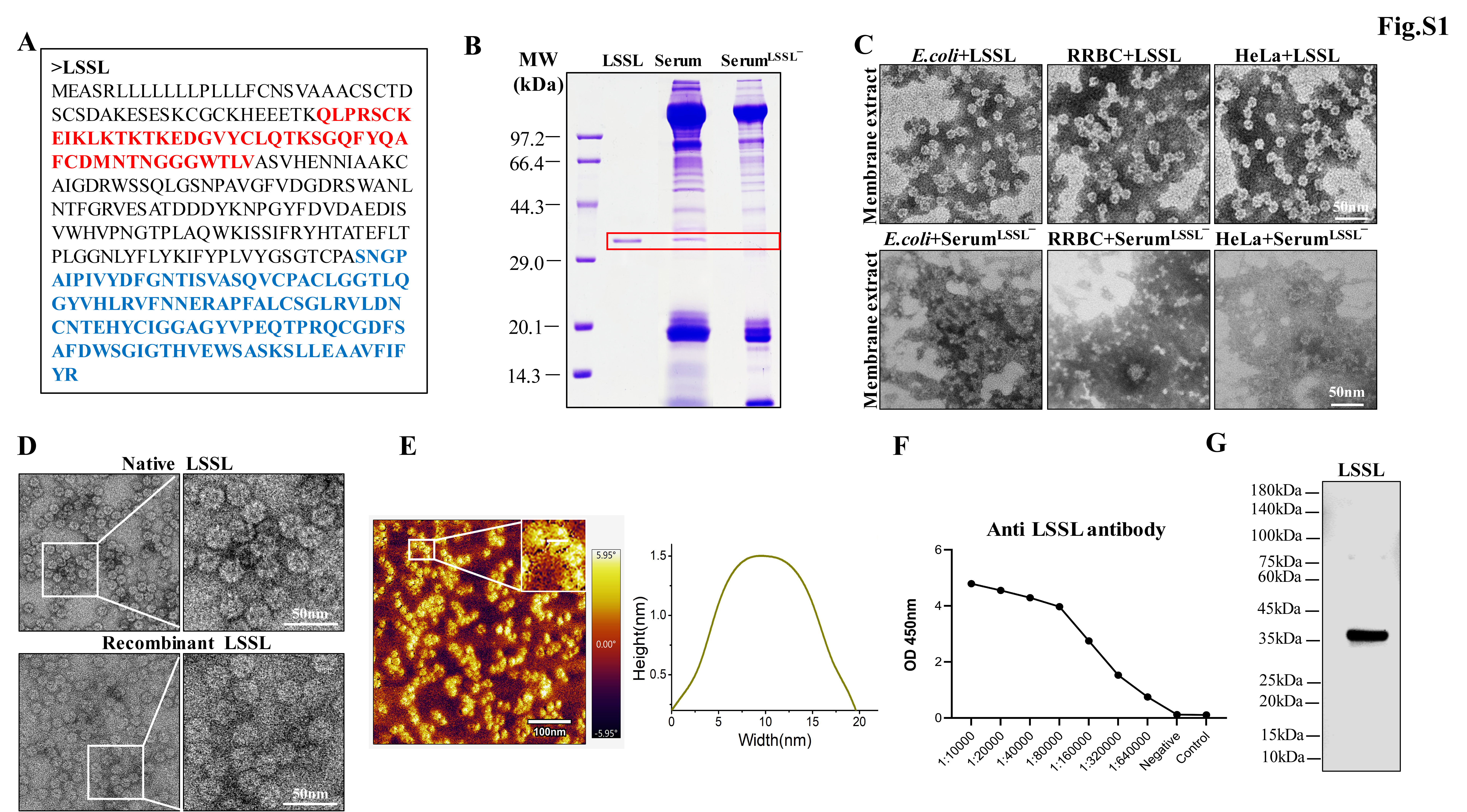

Supplement: Supplementary file 2 — Additional file 2: Fig S1. Discovery of LSSL in lamprey serum. (A) Amino acid sequences of lamprey LSSL. The FReD domain is shown in red and the Intelectin domain is shown in blue. (B) Detection of depletion of LSSL protein in lamprey serum by SDS-PAGE. (C) TEM of membrane protein from E. coli, RRBCs, and HeLa cells treated with LSSL and LSSL-depleted serum. Scale bar, 50 nm. (D) Detection of native and recombinant LSSL protein by TEM. Scale bar, 50 nm. (E) The structure of LSSL as determined by atomic force microscopy. The measurement results show a diameter of 20 nm and a height of 1.5 nm. (F, G) Titer detection of LSSL polyclonal antibodies and detection of specificity by western bloting. [file 11658_2022_401_MOESM2_ESM.tif]

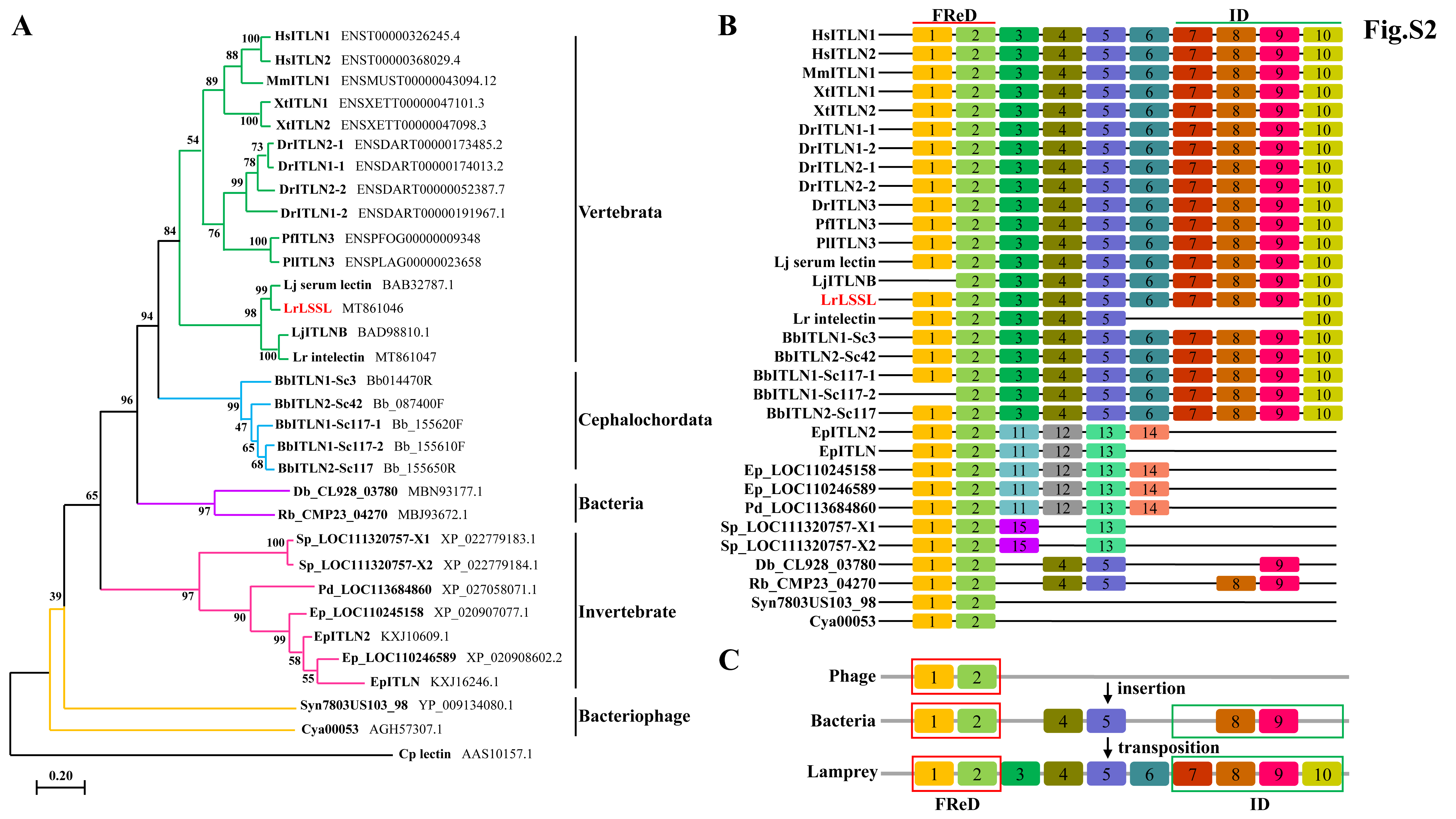

Supplement: Supplementary file 3 — Additional file 3: Fig S2. Origin and evolution of LSSL. (A) The phylogenetic tree for the LSSL family based on the neighbor-joining (NJ) method. An NJ tree was constructed using the amino acid sequences of the LSSL proteins. All species represented by abbreviations are shown in Table S2. The bar (0.020) indicates the genetic distance. Different color lines correspond to different species groups. (B) The motif composition of the LSSL family proteins. (C) Model diagram showing the mechanism of LSSL evolution. [file 11658_2022_401_MOESM3_ESM.tif]

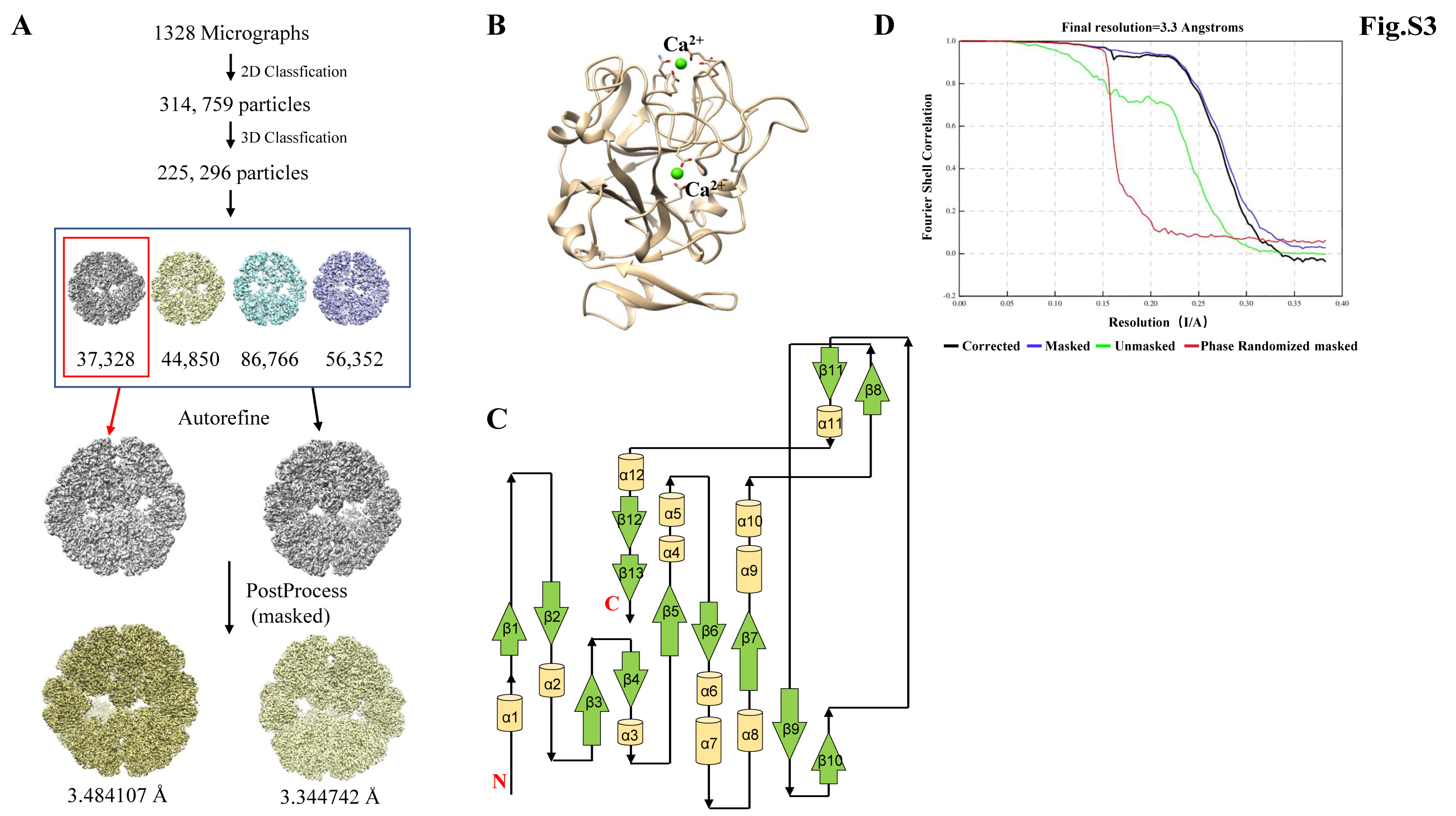

Supplement: Supplementary file 4 — Additional file 4: Fig S3. Cryo-EM analysis. (A) A flow-chart of cryo-EM data processing. (B) Subunit structure in LSSL trimer. (C) Fold diagram for the structure of LSSL. (D) FSC curve (gold standard FSC) as a function of before and after mask application. [file 11658_2022_401_MOESM4_ESM.tif]

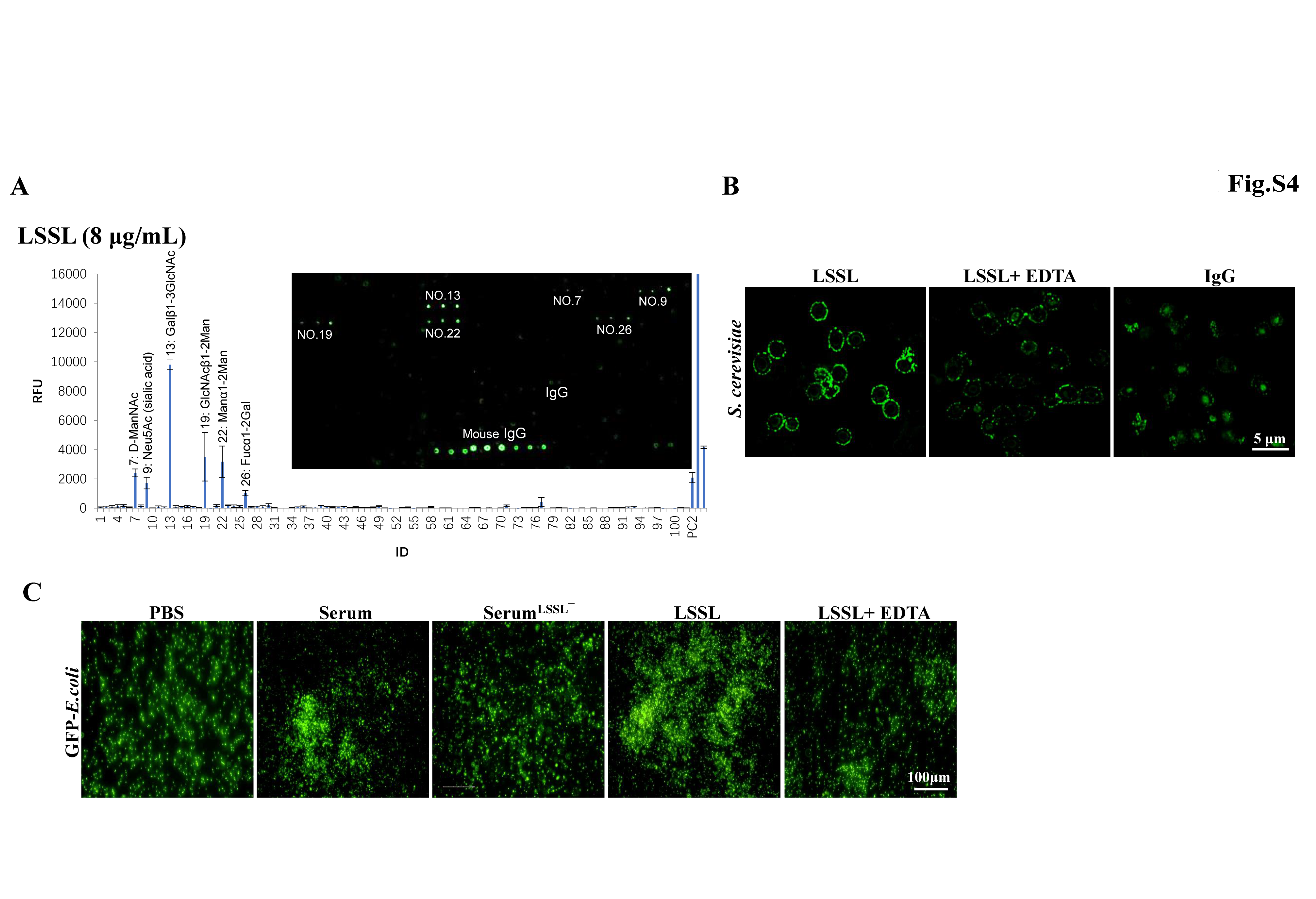

Supplement: Supplementary file 5 — Additional file 5: Fig S4. Glycan selectivity of LSSL assessed by glycan microarrays. (A) LSSL (8 µg/mL) binding to 100 glycan microarray. The concentrations given for the glycan microarray represent those used in the carbohydrate immobilization reaction. Data are presented as the mean ± SDs; (n = 3 technical replicates). (B) Confocal microscopy detection of LSSL binding to yeast. LSSL were incubated with yeast cells (105 cells/well) in PBS for 1 h at room temperature and incubated with FITC-conjugated anti-mouse IgG secondary antibodies followed by confocal microscopy analysis. (C) Quantitative statistical results of GFP-E. coli after high-content screening of bacterial agglutination after LSSL treatment. Bacteria were observed using high-content screening and photographed at the indicated time points (40× magnification). [file 11658_2022_401_MOESM5_ESM.tif]

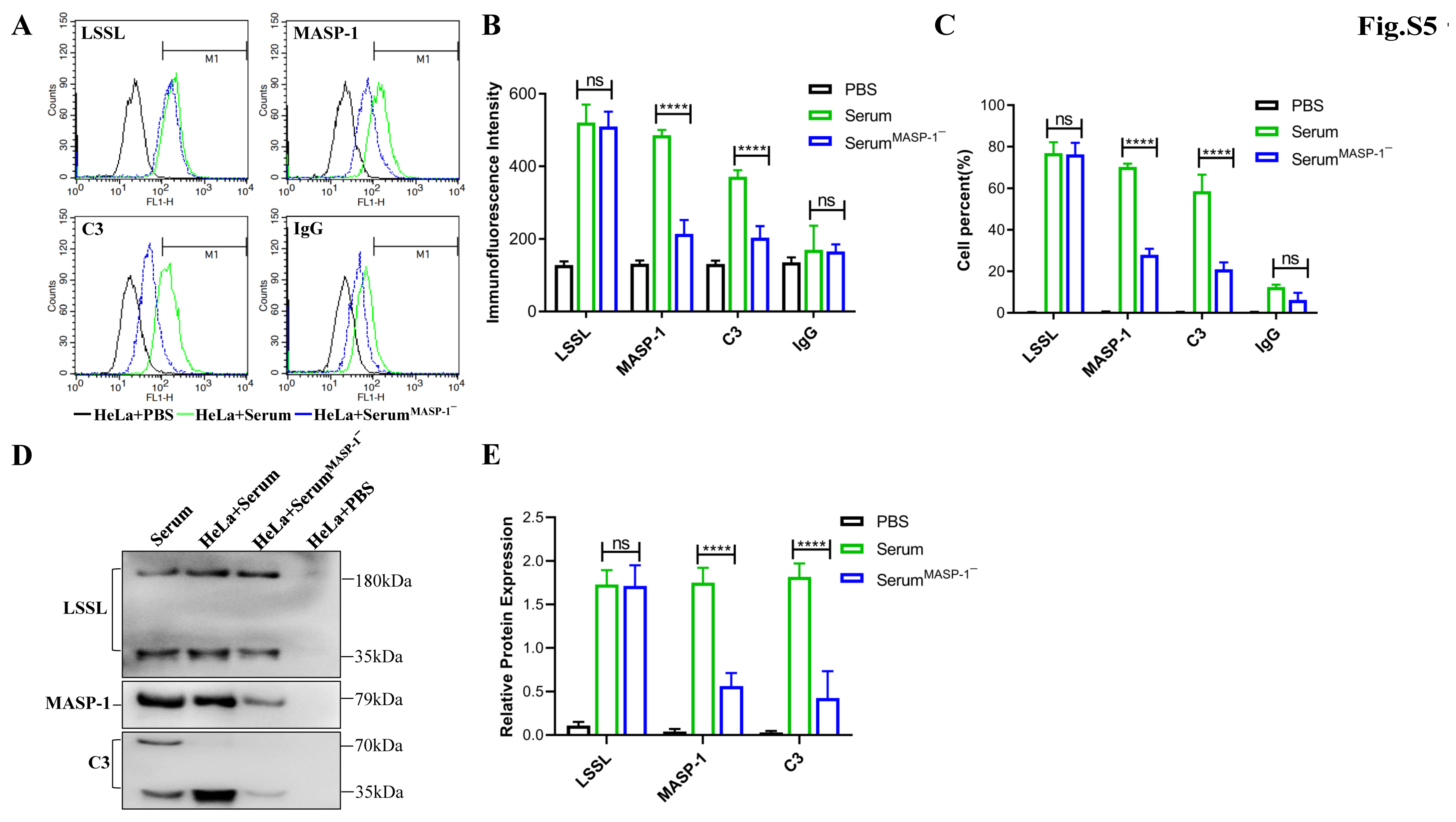

Supplement: Supplementary file 6 — Additional file 6: Fig S5. Elimination of MASP-1 from serum to detect the deposition of LSSL and C3 on the surface of Hela cells. (A) Quantitative analysis of proteins on HeLa cell membranes analyzed by Alexa-488 staining followed by flow cytometry. (B, C) The histogram shows the fluorescence intensity and cell proportions of the above flow cytometry results, respectively. The data are presented as the means ± SDs. (D) Western blotting analysis of depleted and undepleted MASP-1 serum treated HeLa cells, the LSSL, MASP-1, and C3 expression using specific antibodies. (E) Histogram presenting the statistics of the western blotting results. All experiments were repeated at least three times with similar results (n = 3, ****P < 0.0001 ***P < 0.001 **P < 0.01 and *P < 0.05). [file 11658_2022_401_MOESM6_ESM.tif]

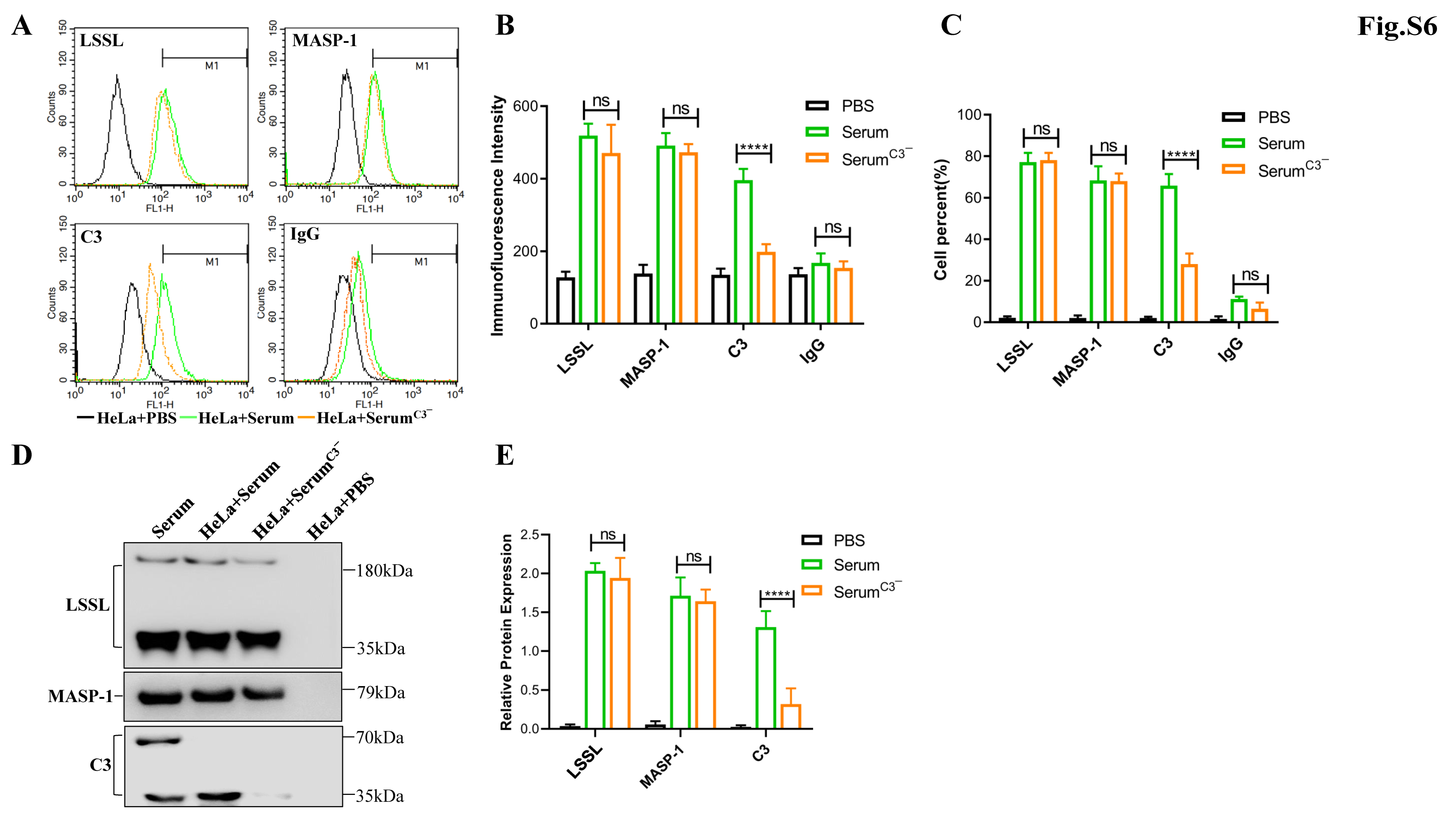

Supplement: Supplementary file 7 — Additional file 7: Fig S6. Elimination of C3 from serum to detect the deposition of LSSL and MASP-1 on the surface of HeLa cells. (A) Quantitative analysis of proteins on the HeLa cells analyzed by Alexa-488 staining followed by flow cytometry. (B, C) The histogram shows the fluorescence intensity and cell proportions of the above flow cytometry results, respectively. The data are presented as the means ± SDs. (D) Western blotting analysis of depleted and undepleted C3 serum treated HeLa cells, the LSSL, MASP-1, and C3 protein expression using specific antibodies. (E) A histogram showing the statistics of the western blotting results. All experiments were repeated at least three times with similar results (n = 3, ****P < 0.0001 ***P < 0.001 **P < 0.01 and *P < 0.05). [file 11658_2022_401_MOESM7_ESM.tif]

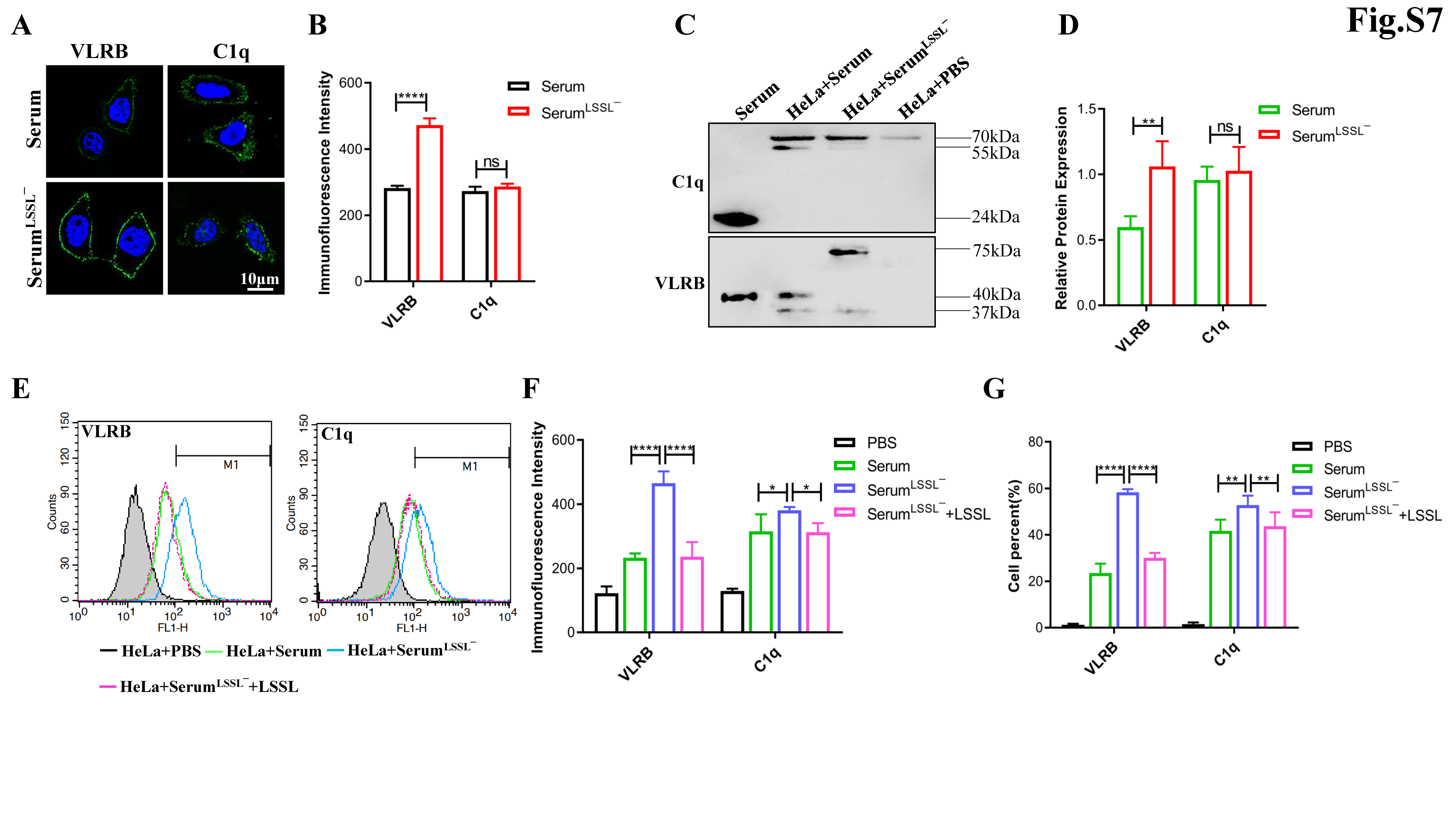

Supplement: Supplementary file 8 — Additional file 8: Fig S7. Elimination of LSSL from serum to detect the deposition of VLRB and C1q on the surface of HeLa cells. (A) Immuno-fluorescence detection of VLRB and C1q deposited on the surface of HeLa cells treated with lamprey serum and LSSL-depleted serum. Scale bars, 10 µm. (B) A histogram shows the statistics of the above-mentioned results. The data are presented as the means ± SDs. (C) Western blotting analysis with mouse anti-VLRB monoclonal antibodies [34] and rabbit anti-C1q [34]. (D) A histogram showing the statistics of the western blotting results. All experiments were repeated at least three times with similar results. (E) Quantitative analysis of proteins on the cell analyzed by Alexa-488 staining followed by flow cytometry. (F, G) The histogram shows the fluorescence intensity and cell percentage statistics of the above flow cytometry results, respectively. The data are presented as the means ± SDs (n = 3, ****P < 0.0001 ***P < 0.001 **P < 0.01 and *P < 0.05). [file 11658_2022_401_MOESM8_ESM.tif]
